# A ‘three-axis synergy’ immunotherapeutic strategy for malignant bone tumors based on natural bioactives and bioactive materials

**DOI:** 10.1016/j.bioactmat.2026.04.031

**Published:** 2026-04-29

**Authors:** Xiangjun Pan, Ruiyan Li, Xinyu Xu, Zehao Yu, Shibo Liu, Dapeng Zeng, Wenrui Qu, Zhicheng Zhang, Hao Wang, Yanguo Qin

**Affiliations:** a Department of Joint Surgery of Orthopaedic Center, The Second Hospital of Jilin University, Changchun, 130041, People's Republic of China; b Joint International Research Laboratory of Ageing Active Strategy and Bionic Health in Northeast Asia of Ministry of Education, Jilin University, Changchun, Jilin Province, People's Republic of China; c Department of Plastic Surgery, Beijing Tsinghua Changgung Hospital, School of Clinical Medicine, Tsinghua Medicine, Tsinghua University, People's Republic of China

**Keywords:** Immunotherapy, Bioactive materials, Natural bioactives, Malignant bone tumor, Immune evasion

## Abstract

This review presents an innovative "three-axis synergy" immunotherapeutic strategy for malignant bone tumors, combining natural bioactives with bioactive materials to overcome the challenges posed by the immunosuppressive microenvironment (IME) of tumors. Malignant bone tumors create an IME characterized by immune evasion, bone destruction, and physicochemical dysregulation, which limits the effectiveness of conventional therapies. Natural bioactives, known for their immunomodulatory effects, can potentially reprogram the IME, but their clinical application is hindered by low bioavailability and limited targeting efficiency. Bioactive materials, such as scaffolds and nanoparticles, can improve the delivery, retention, and targeted release of these bioactives, while also supporting bone regeneration. By combining natural bioactives with bioactive materials, this synergistic approach offers a dual-functional therapeutic platform that not only targets immune suppression but also promotes bone repair. This strategy addresses the key barriers of the IME, including immune imbalance, bone resorption, and physicochemical abnormalities. Although challenges remain in the clinical translation of these natural bioactives, their integration with bioactive materials provides a promising direction for improving treatment outcomes of malignant bone tumors. This approach offers new insights into the future of cancer immunotherapy and bone regeneration.

## Highlights

• Proposes a "three-axis synergy" immunotherapy strategy for bone tumors, integrating bone regeneration, physicochemical property rebalancing, and immune modulation into one coherent framework.
• Highlights the synergistic effects of natural bioactives and biomaterials in reshaping the bone tumor microenvironment, rather than treating antitumor therapy and tissue repair as separate processes.
• Discusses translational design principles, potential clinical combinations, and current challenges, providing practical insights for researchers and clinicians in the field.

## Graphical abstract

**Figure:**
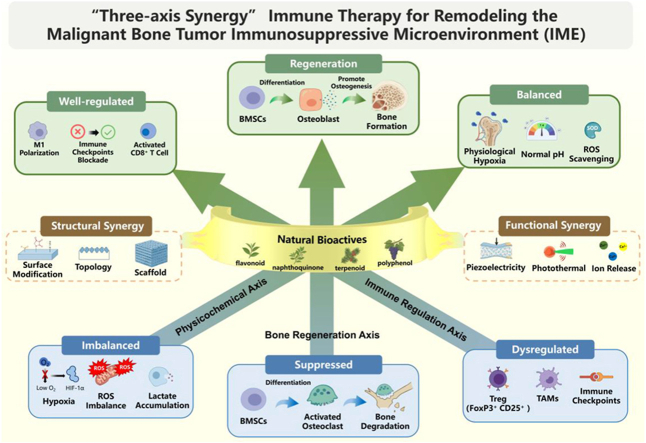

## 1 Introduction

Malignant bone tumors pose a serious threat to patient survival and quality of life [[1], [2], [3], [4]]. In recent years, immunotherapeutic strategies represented by immune checkpoint inhibitors and chimeric antigen receptor T-cell (CAR-T) therapy have introduced new avenues for the treatment of malignant bone tumors [[5], [6], [7]]. However, the overall efficacy of current immunotherapies in malignant bone tumors remains limited. A key reason is that this limitation is attributable to the persistent interference of the immunosuppressive microenvironment (IME), which hinders local drug delivery, tissue penetration, and the establishment of effective immune surveillance [8,9]. Therefore, overcoming the therapeutic barriers imposed by the IME has become a central challenge in improving the efficacy of immunotherapy for malignant bone tumors. The three core features of the IME in malignant bone tumors are immunosuppression, bone destruction, and physicochemical dysregulation. First, along the immunoregulatory axis, the enrichment of immunosuppressive cells within the IME, the exhaustion of effector T cells, and the failure of local immune surveillance collectively promote tumor immune evasion and therapeutic resistance [8,10]. Second, along the physicochemical remodeling axis, malignant bone tumor tissues are accompanied by abnormalities such as hypoxia, acidosis, and redox imbalance. These physicochemical disturbances not only reduce the efficiency of local drug delivery, but also create conditions more conducive to tumor progression and the maintenance of immunosuppression [11,12]. Third, along the bone regeneration axis, the IME of malignant bone tumors disrupts the dynamic balance between local bone formation and bone resorption, leading to pathological bone loss, structural bone destruction, and limited postoperative repair of bone defects [[12], [13], [14]].

These three axes do not operate independently. Instead, they form a self-reinforcing loop: immunosuppression weakens antitumor surveillance, bone destruction facilitates invasion and recurrence, and physicochemical abnormalities impair drug delivery, immune function, and bone regeneration [11,[15], [16], [17]]. Therefore, the limited efficacy of immunotherapy for malignant bone tumors cannot be attributed solely to immunosuppression, but rather reflects therapeutic barriers jointly driven by immune imbalance, physicochemical dysregulation, and disruption of bone homeostasis. Single-modality interventions are often insufficient to correct abnormalities across all three axes simultaneously. Accordingly, there is an urgent need to develop a “three-axis synergy” therapeutic strategy that integrates immune modulation, physicochemical remodeling, and bone regeneration.

In the context of “three-axis synergy,” bioactive materials have demonstrated important advantages. In recent years, a wide range of materials, including inorganic nanomaterials, natural and synthetic polymers, biologically derived scaffolds, and biomimetic delivery systems, have shown potential in the treatment of malignant bone tumors [15,18,19]. On the one hand, bioactive materials can regulate cellular behavior within the IME through their intrinsic bioactivity and surface topographical features, thereby inhibiting tumor progression while promoting the repair of bone defects [12,[20], [21], [22]]. On the other hand, some materials can be combined with physical therapeutic modalities, such as photothermal, photodynamic, sonodynamic, and magnetic hyperthermia therapies, to enhance tumor killing and increase the immunogenicity of tumor cells, thereby producing immunosensitizing effects [23,24]. In addition, when used as delivery vehicles, bioactive materials can improve therapeutic efficiency by increasing local drug accumulation and enhancing tissue-specific delivery [16,25]. At the same time, however, bioactive materials also have certain limitations. For example, materials that rely on metal ions for their function often face biosafety challenges within the effective concentration range, whereas therapies dependent on exogenous physical stimulation may be constrained by insufficient tissue penetration and potential long-term adverse effects. Therefore, reliance on material platforms alone remains insufficient to comprehensively address the full spectrum of abnormalities within the IME.

Moreover, natural bioactives have attracted sustained attention because of their immunomodulatory potential. Studies have shown that a variety of natural bioactives can inhibit tumor proliferation, invasion, and metastasis by regulating signaling pathways such as EGFR/Src, and can also modulate immune–related signaling pathways to some extent, thereby contributing to the remodeling of the IME in malignant bone tumors [26,27]. In addition, natural bioactives possess advantages such as multi-target immunomodulatory effects and potential in regulating bone metabolism [[28], [29], [30]]. However, their translation is constrained by limited targeting efficiency and bioavailability, and thus they are more suitable for use in combination with biomaterials.

**Fig. 1:**
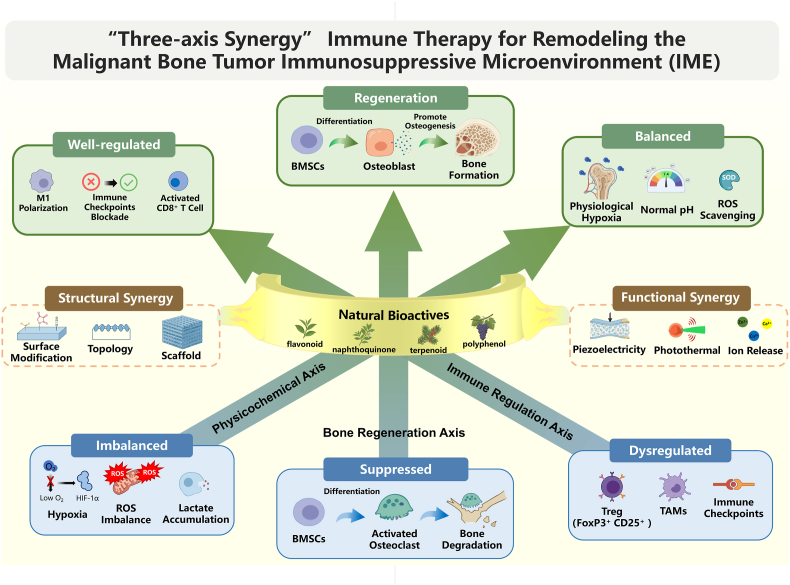
The IME of malignant bone tumors is characterized by imbalances in physicochemical properties (e.g., ROS accumulation and acid buildup), suppression of bone regeneration accompanied by enhanced bone destruction, and immune dysregulation. Natural bioactives and bioactive materials, through synergistic interactions in both structure and function, hold promise to fundamentally transform the immunosuppressive state of the IME in malignant bone tumors via a “three-axis synergy” involving immunomodulation, bone regeneration, and physicochemical microenvironment remodeling.

Combining natural bioactives with bioactive materials may address the IME more effectively than either strategy alone. Biomaterials improve delivery, retention, targeting, and bone repair, whereas natural bioactives provide complementary immunomodulatory effects across immune regulation, physicochemical remodeling, and bone regeneration. On this basis, the synergistic application of natural bioactives and bioactive materials may represent an emerging “three-axis synergy” strategy for the immunotherapy of malignant bone tumors, providing a new theoretical foundation for overcoming current bottlenecks in both therapeutic efficacy and postoperative functional reconstruction (Fig. 1).

## 2 Comparison between bone and malignant bone tumor microenvironments

The bone microenvironment refers to the local milieu within bone tissue surrounding osteocytes and the bone marrow cavity. It is jointly constituted by diverse cellular components (including osteoblasts and osteoclasts), the extracellular matrix, vasculature, nerves, cytokines, and the prevailing mechanical and metabolic conditions. Through dynamic interactions among these elements, the bone microenvironment regulates bone formation, bone resorption, bone remodeling, and hematopoiesis (Table 1).

**Table 1:** The IME characteristics of malignant bone tumors.

Axis	Characteristics	Outcomes	Ref.
**Physicochemical properties**	Aberrant vascularization and hypoxia	Promotes hypoxic adaptation; Metabolic reprogramming	[[55], [56], [57]], [87]
	Glycolysis and acidic accumulation	Lactate accumulation and decreased extracellular pH	[[55], [56], [57], [58], [59]]
	Maintenance of the pH gradient	Enhances invasive potential	[58], [59]
	ROS accumulation and redox adaptation	Moderate ROS acts as a signaling cue for hypoxic adaptation and survival; Excessive ROS induces oxidative damage;	[[63], [64], [65]], [90]
	Direct regulation of bone-cell behavior	Contributes to impaired bone regeneration, enhanced bone resorption	[[87], [88], [89], [90]]
**Immunomodulatory**	Immune checkpoint suppression	Stable T-cell reactivation is difficult to achieve; PD-1/PD-L1 monotherapy shows limited efficacy.	[[66], [67], [68]]
	Compensatory exhaustion pathway	Weakens the response to single checkpoint inhibition; Supports combination immunotherapy strategies.	[69], [70]
	Reinforcement of adaptive and innate immune escape	Strengthens immune escape at both adaptive and innate immune levels	[[71], [72], [73], [74], [75], [76], [77], [78]]
	Enrichment of immunosuppressive cells	Maintains an immunosuppressive microenvironment	[[80], [81], [82], [83], [84]]
	Immune reprogramming by metabolism	Further reinforces immune tolerance and immune evasion in malignant bone tumors.	[[60], [61], [62]]
**Bone regeneration/destruction**	Resorption-dominant imbalance	Leads to bone loss dominated by bone resorption.	[85], [86]
	Positive feedback between matrix degradation and tumor progression	Forms a positive feedback loop between bone destruction and tumor progression.	[85], [86]
	Pro-resorptive effects of acidosis and ROS	Amplifies inflammatory and bone-resorption signals while suppressing osteogenesis.	[[88], [89], [90]]
	Bone destruction driven by immune suppression	Accelerates bone resorption and facilitates tumor progression.	[90], [91]

**Fig. 2:**
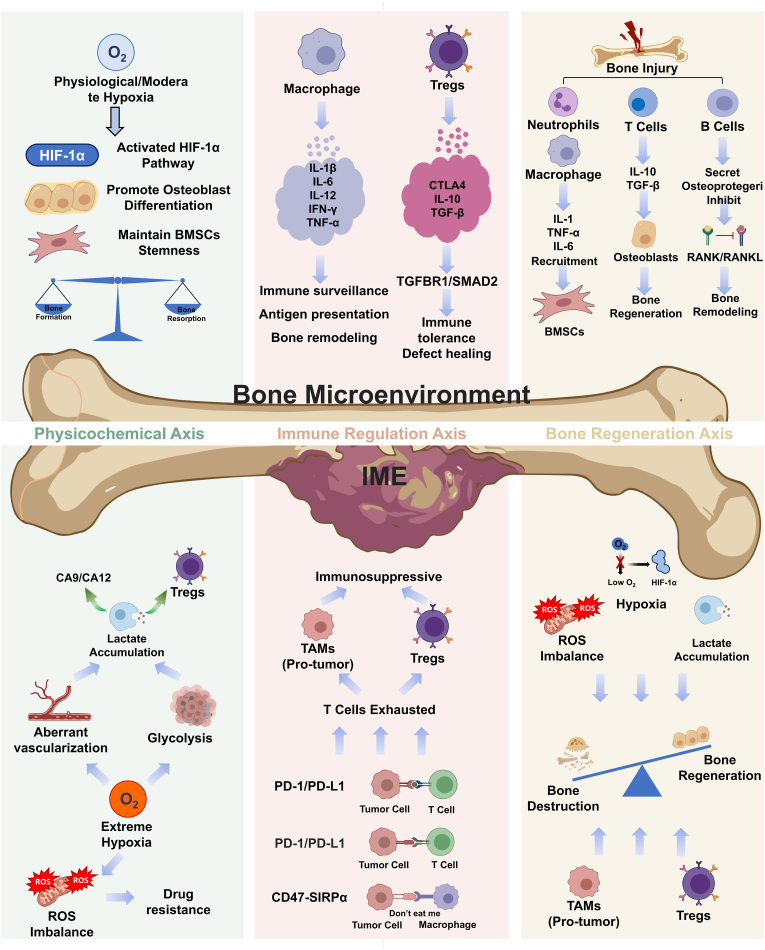
Comparison between normal bone tissue and the IME of malignant bone tumors. Normal bone tissue is maintained by the coordinated balance of three interrelated axes, including immune regulation, physicochemical regulation, and bone remodeling/regeneration. Under physiological conditions, the relatively hypoxic state of bone tissue supports normal bone homeostasis, while resident immune cells, including Tregs and myeloid cells, help preserve immune tolerance and immune surveillance in a balanced manner. In malignant bone tumors, this three-axis balance is disrupted. On the physicochemical axis, rapid tumor cell proliferation aggravates hypoxia and other microenvironmental abnormalities. On the immune axis, malignant cells promote immune escape through the combined action of immune checkpoints and immunosuppressive cells. On the bone regeneration axis, these changes impair the balance between bone resorption and bone formation, resulting in reduced bone regenerative capacity and progressive bone destruction.

Understanding the distinctions between the normal bone microenvironment and the IME of malignant bone tumors is important for the development of effective immunotherapies. In the bone microenvironment, immune cells such as mononuclear macrophages, T cells, and NK cells perform distinct functions to maintain local metabolic homeostasis, a suitable acid-base balance, and the equilibrium between osteogenesis and osteoclastogenesis. These cells play crucial roles in various processes including bone growth and differentiation, callus formation, and bone remodeling. They defend against tumor invasion and pathogens through antigen recognition and cell-mediated cytotoxicity, while also secreting various cytokines to modulate immune responses within the microenvironment (Fig. 2).

However, in the IME of malignant bone tumors, the rapid proliferation of tumor cells and their unique mechanisms of immune checkpoint molecule expression often disrupt the normal function of immune cells. This leads to a breakdown in bone metabolic homeostasis and transforms the local microenvironment into an immunosuppressive niche that favors tumor survival and progression.

### 2.1 Bone microenvironment

The bone microenvironment is a complex and dynamically balanced physiological system composed of various cell types, extracellular matrix, abundant bioactive factors, vascular and neural networks, as well as unique physicochemical properties (such as pH levels) [31,32]. Under physiological conditions, it regulates bone formation, resorption, and remodeling through orchestrated signaling networks. In pathological states, however, its dysregulation is closely associated with the development and progression of orthopedic diseases, including malignant bone tumors.

#### 2.1.1 Physicochemical characteristics and immunological properties

Bone tissue exists in a physiologically hypoxic environment [33], and moderate hypoxia is essential for the normal differentiation and function of osteoblasts [34,35]. Hypoxia activates the HIF-1α (hypoxia-inducible factor-1α) pathway, a central regulator of cellular adaptation to low oxygen conditions. However, severe or chronic hypoxia suppresses osteoblast function and reduces bone formation. Meanwhile, osteoclast precursors exhibit enhanced differentiation and activation under hypoxic conditions [36,37]. A low-oxygen environment helps maintain mesenchymal stem cells (MSCs) in an undifferentiated state, preserving their "stemness" and self-renewal capacity [38]. When osteogenic differentiation is required, HIF-1α-mediated signaling serves as a critical regulatory mechanism for tissue-resident stem cells. This intricate interplay underscores the importance of both HIF-1α signaling and appropriately maintained hypoxia in regulating the balance between bone formation and resorption.

In addition to the dynamic balance between osteoblasts and osteoclasts, immune cells derived from hematopoietic stem cells (HSCs), such as T cells, macrophages, and monocytes, also play crucial roles in shaping and maintaining bone microenvironment homeostasis. Among these, macrophages dynamically adapt their function and polarization phenotype in response to environmental cues. They contribute to inflammatory responses through the release of cytokines including IL-1β, IL-6, IL-12, IFN-γ, and TNF-α [39], thereby facilitating immune surveillance, antigen presentation, and promoting bone repair and remodeling following injury [40]. Regulatory T cells (Tregs) participate in tissue repair and immune homeostasis through signaling molecules such as IL-10, TGF-β, and CTLA-4, as well as by regulating the state of macrophages [41,42].

Furthermore, a multitude of cytokines within the bone microenvironment play crucial roles in maintaining metabolic homeostasis and regulating immune responses. Angiogenic factors such as VEGF and osteogenic factors like BMP-2 provide nutritional support and promote osteogenesis during bone development, injury repair, and remodeling processes [43]. The dynamic balance between bone formation and resorption is regulated through the interplay of pro-inflammatory cytokines, including TNF-α, IL-1, IL-6, and IFN-γ, and anti-inflammatory mediators such as IL-4 and IL-10 [44]. In the malignant bone tumor microenvironment, however, the expression of anti-inflammatory cytokines is further upregulated. This enhanced immunosuppression dampens both adaptive and innate immune responses against tumor cells, ultimately facilitating immune escape and tumor progression.

#### 2.1.2 Immunoregulatory–osteoregenerative interactions

There exists a dynamic balance between bone formation and bone resorption within the bone microenvironment, involving osteoblasts, osteoclasts, and other bone-related cells; immune cells such as macrophages and Tregs; and an immune-related signaling network. Through coordinated actions involving cell–cell interactions, cytokines/chemokines, and exosomes, these components orchestrate the interplay between immune regulation and bone regeneration, which serves as a core mechanism for maintaining bone homeostasis and facilitating tissue repair [45,46].

During bone injury, the hematoma and the local inflammatory microenvironment rapidly recruit innate immune cells, such as neutrophils and macrophages [[47], [48], [49]]. These cells secrete pro-inflammatory cytokines, including IL-1, TNF-α, and IL-6, which not only facilitate the clearance of necrotic tissue and pathogens but also provide directional cues for the recruitment and proliferation of MSCs [50,51]. Adaptive immune cells contribute to the regulation of this process: for example, certain subsets secrete IL-10 and TGF-β to restrain excessive inflammation and promote osteogenic differentiation of MSCs. In contrast, effector T cell populations produce cytokines such as IFN-γ and IL-17, which, depending on the stage and local context, modulate osteoclast activity and osteogenic signaling pathways, thereby influencing bone matrix remodeling. B cells further participate in skeletal homeostasis by secreting osteoprotective factors, including osteoprotegerin (OPG), and regulating the RANK/RANKL/OPG axis, thus affecting the balance between bone resorption and formation [46]. In addition, the cytokine interaction network among immune cells plays a critical role in bone regeneration. Macrophage colony-stimulating factor (M-CSF) and RANKL are essential for osteoclast differentiation, whereas TGF-β and bone morphogenetic proteins (BMPs) promote osteogenesis [45].

Taken together, immune regulation and bone regeneration within the physiological bone microenvironment constitute a multilayered and dynamically coordinated process. Immune cells are involved not only in the initiation of inflammation and immune clearance but also in the orchestration of bone repair through cytokine-mediated crosstalk with bone cells. A deeper understanding of the mechanisms underlying immune–bone regenerative interactions will help elucidate the principles governing skeletal homeostasis and provide a theoretical basis for the development of immunomodulation-based bone regenerative strategies, including immunoregulatory biomaterials and cell-based therapies [[52], [53], [54]].

### 2.2 IME of malignant bone tumors

Compared with the microenvironment of normal bone tissue, the IME of malignant bone tumors exhibits distinct physicochemical properties, characteristic patterns of immune activity, and an imbalance between bone regeneration and bone destruction.

#### 2.2.1 Physicochemical properties of the IME in malignant bone tumors

The IME of malignant bone tumors is characterized by chronic hypoxia, acidosis, and ROS accumulation. These abnormalities arise from restricted perfusion and disrupted metabolic homeostasis. Abnormal vasculature, low perfusion, and high oxygen demand together sustain hypoxia and activate HIF-1α-related programs. In this context, hypoxia-induced pathways such as HIF-1α are continuously activated, driving enhanced glucose uptake and glycolysis, and upregulating genes associated with acid-base regulation (SLC16A3/MCT4), angiogenesis (VEGF/VEGF-A), and invasion (CXCR4, MMP2, MMP9) [[55], [56], [57]].

Increased hypoxia and glycolysis drive continuous lactate and H^+^ production, whereas low perfusion limits their clearance. As a result, extracellular pH decreases. Tumor cells further maintain this pH gradient through carbonic anhydrases such as CA9 and CA12, which supports survival and invasion [58,59]. The acidic microenvironment and accumulation of lactate metabolic products alter the functional state of surrounding cells, for instance, by inhibiting the metabolism and activation of various effector immune cells [60]. In the tumor IME, the differentiation and immunosuppressive function of Tregs are promoted by the acidic microenvironment, thereby facilitating the formation of the malignant bone tumor IME [61,62].

**Fig. 3:**
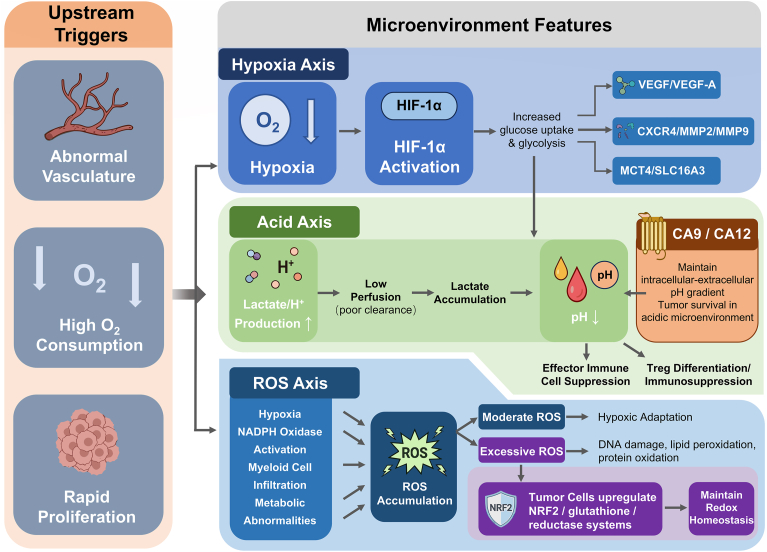
The rapid proliferation of malignant bone tumor cells and their aberrant cytokine secretion lead to abnormal vascularization and hypoxia within the tumor microenvironment. Under these conditions, HIF-1α and its downstream pathways are activated, promoting the overexpression of cytokines that facilitate tumor cell invasion. Tumor cells also accelerate lactate accumulation in the microenvironment through glycolysis, thereby creating an acidic microenvironment that promotes bone destruction. In addition, the efficiency of ROS clearance is reduced, further contributing to bone tissue damage.

A hypoxic state does not imply a reduction in ROS. On the contrary, under hypoxic conditions, mitochondria are more prone to generating superoxide anions and hydrogen peroxide [63,64]. Additionally, tumor cell metabolic abnormalities, activation of NADPH oxidase, and myeloid cell infiltration can further increase the ROS burden. Moderate levels of ROS act as signaling molecules to promote hypoxic adaptation and cell survival pathways, whereas excessive ROS can trigger DNA damage, lipid peroxidation, and protein oxidation, ultimately hindering cell survival. Therefore, tumor cells often compensate by upregulating antioxidant transcriptional networks such as NRF2, the glutathione system, and related reductase systems, in order to maintain redox homeostasis and enhance drug resistance/tolerance phenotypes [65] (Fig. 3).

#### 2.2.2 Immunomodulatory characteristics of the IME in malignant bone tumors

In the IME of malignant bone tumors, the PD-1/PD-L1 axis forms the foundation of immune suppression [66,67]. Furthermore, the IME is characterized by MHC-I loss and insufficient antigen presentation, leading to inadequate immune activation. As a result, even blocking the PD-1 axis fails to produce stable T cell reactivation, which is consistent with multiple studies showing limited efficacy of PD-1/PD-L1 monotherapy, such as the lack of clear clinical effects of pembrolizumab in osteosarcoma [68]. The insufficient therapeutic effect of PD-1/PD-L1 axis blockade also suggests the presence of other immune escape mechanisms in the IME. TIM-3/Gal-9 is often considered a signaling pathway that can sustain immune suppression even after PD-1 blockade, with evidence of a PD-1-independent exhaustion mechanism [69,70]. Although much of this research comes from non-bone tumors, it offers valuable insights into the low response of osteosarcoma to PD-1 monotherapy.

However, osteosarcoma-specific functional evidence remains relatively scarce, and careful extrapolation is required. Additionally, B7-H3 is often highly expressed in osteosarcoma. Its effects extend beyond T-cell suppression and are associated with proliferation, invasion, metastasis, metabolic reprogramming, angiogenesis, and therapeutic resistance, and it has entered more clinically relevant translational pathways, such as the in *vitro* and in *vivo* validation of CAR-T therapies targeting B7-H3 in osteosarcoma [[71], [72], [73]]. However, the role of B7-H3 in immune function within different tumor microenvironments remains controversial, and the efficacy of drugs targeting this immune checkpoint still requires further clinical validation [74].

Moreover, CD47-SIRPα represents a "don't eat me" signal that directly reduces macrophage phagocytosis and antigen presentation efficiency. The expression of CD47 in osteosarcoma is associated with tumor metastasis and escape from phagocytosis [[75], [76], [77]]. CD47 represents an important nexus linking innate immune evasion with impaired adaptive antitumor immunity. Its therapeutic potential mainly lies in relieving macrophage inhibition, restoring phagocytic activity, and enhancing the antitumor immune cascade. At the same time, however, its broad expression across normal tissues poses a major challenge for CD47-targeted therapy. The limited efficacy observed in first-generation CD47-targeted programs does not necessarily indicate that this pathway is ineffective, rather, it suggests that its clinical development requires a more precise strategy, including optimization of targeting molecule design, reduction of off-tumor toxicity, and improvement of biomarker-based patient selection [78,79].

In summary, the PD-1/PD-L1 axis provides the basic immune suppressive activity in malignant bone tumors, TIM-3/Gal-9 offers compensatory immune exhaustion after PD-1 blockade, and B7-H3 and CD47 strengthen immune escape at adaptive and innate immune levels, collectively contributing to the insensitivity of bone tumors to monotherapy with a single checkpoint inhibitor.

Under the influence of immune checkpoints, immunosuppressive cells become enriched in the IME of malignant bone tumors. Tumor-associated macrophages (TAMs) differentiate within the IME toward a pro-tumor phenotype. In previous studies, macrophage polarization phenotypes were simplistically divided into M1 and M2 types; however, current findings indicate that this classification is no longer sufficient to guide immunotherapy for malignant bone tumors. Although M2 macrophages can be broadly summarized as pro-tumorigenic, they comprise multiple subtypes and participate in diverse biological processes within the tumor microenvironment, including angiogenesis and invasion [80,81]. Current research suggests that macrophages in the malignant bone tumor IME may exhibit a continuously varying polarization spectrum. A cohort study of osteosarcoma provides support for this view, the study identified C1Q^+^ TAMs as a myeloid population with a significant proportion in osteosarcoma and found an association with improved survival [82]. This also suggests that, even among TAMs, different subpopulations may imply distinctly different biological and prognostic significance. The expansion of Tregs in the IME is also not an isolated event, rather, it results from the combined effects of immune checkpoints (e.g., PD-1, TIM-3) inducing effector T cells into a hyporesponsive or exhausted state [83], and the joint influence of tumor metabolism and the cytokine milieu. Single-cell transcriptomic data from osteosarcoma show that Tregs are highly infiltrated in the samples and exhibit metabolic/functional features characterized by activation of pathways such as oxidative phosphorylation, angiogenesis, and mTORC1 signaling [84]. The coordinated actions of Tregs and TAMs contribute to maintaining an immunosuppressive state in the IME.

#### 2.2.3 Imbalance between bone regeneration and bone resorption

In malignant bone tumors, bone remodeling shifts toward destruction under the combined influence of physicochemical stress and immune dysfunction. Tumor cells and immunosuppressive populations promote osteoclastogenesis through pathways such as RANK/RANKL/OPG while suppressing osteoblast differentiation and mineralization. This ultimately results in bone loss dominated by bone resorption [85,86]. The degradation of the bone matrix releases various pro-tumor factors, which in turn promote tumor survival, invasion, and immune evasion, forming a positive feedback loop of tumor progression and bone loss. In this process, the physicochemical characteristics of the IME, including hypoxia, acidity, and increased ROS levels, are not merely metabolic by-products but directly participate in the regulation of bone cell differentiation and biological behavior. Hypoxia-related pathways and the status of immune cells in osteosarcoma are directly associated with treatment efficacy and prognosis [87]. The acidic microenvironment can promote osteoclast activity, enhancing osteoclast formation and the transcription of genes related to bone resorption while inhibiting the osteogenic process, leading to bone loss [88,89]. ROS and the stress responses it induced can promote dysfunction in bone-related cells and amplify inflammation and bone resorption signals [90].

The local immune characteristics have a bidirectional effect on bone remodeling and tumor progression: on one hand, the immunosuppressive state (such as the immunosuppressive phenotype of TAMs, low T-cell responsiveness, and T-cell exhaustion) can weaken anti-tumor immunity and induce T cells to produce pro-osteoclastogenic signals (e.g., RANKL, TNF), thus promoting bone resorption and tumor expansion [90,91]. On the other hand, when T cells are effectively activated, the specific cholinergic/Th1-like signals they produce can inhibit osteoclastogenesis and enhance anti-tumor effects, suggesting that anti-tumor activity may also have the potential to counteract bone destruction under certain conditions [90,92]. In addition, recent evidence suggests that the balance between effector T cells (Teffs) and Tregs may serve as an important indicator of immune functional status, as a reduced Teff/Treg ratio is generally associated with enhanced immunosuppression and weakened antitumor immunity; although this concept has been discussed primarily in pancreatic cancer, it provides a valuable framework for understanding how T cells disequilibrium may contribute to immune escape and IME remodeling in malignant bone tumors [93].

Moreover, single-cell and spatial omics studies of the osteosarcoma IME suggest that tumor cells can interact with myeloid cells, T/NK cells, and osteoblastic lineage cells, reshaping the local niche. Different TAM subgroups may correspond to distinct immune and prognostic outcomes, implying that TAMs do not solely promote tumor progression, but rather their effects depend on subgroup composition and functional status [[94], [95], [96]]. Therefore, the imbalance between bone regeneration and bone destruction in the malignant bone tumor IME should not be simplified as a result of enhanced osteoclast activity and suppressed osteogenesis, but rather viewed as the outcome of the combined influence of physicochemical properties (e.g., hypoxia, acidity, ROS) and pro-tumor/anti-tumor inflammatory signals. Consequently, intervention strategies are likely to require concurrent consideration of physicochemical regulation, immune activity modulation, and promotion of bone remodeling.

## 3 Natural bioactives reprogram the IME in malignant bone tumors

**Fig. 4:**
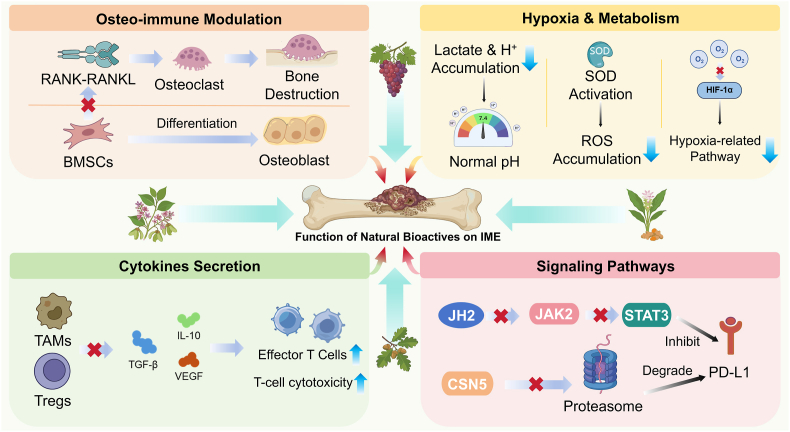
An immunotherapeutic strategy based on natural bioactives. Natural bioactives promote the ubiquitin-mediated degradation of PD-L1 and inhibit immunosuppressive cell populations from producing cytokines such as IL-10, thereby restoring immune surveillance. They also suppress the RANK-RANKL axis to regulate osteoclast differentiation and promote the osteogenic differentiation of BMSCs, thereby reestablishing the balance between bone regeneration and bone resorption. In addition, they modulate the three major physicochemical properties of the IME. Ultimately, these effects collectively enable comprehensive remodeling of the IME in malignant bone tumors.

Natural bioactives can remodel the IME of malignant bone tumors by regulating the physicochemical properties of the local microenvironment, the expression levels of immune checkpoint molecules, immune cell functions, and the processes of bone regeneration. While promoting bone regeneration, these compounds may also improve immune surveillance and enhance immune responses that contribute to tumor control (Table 2). However, the clinical translation of natural bioactive compounds still faces several challenges, particularly with respect to bioavailability and tissue specificity, which require further investigation and resolution (Fig. 4).

**Table 2:** Natural bioactives against the IME of malignant bone tumors.

Characteristics		Natural bioactives	Outcomes	Reference
**Physicochemical properties**	**Hypoxia pathway modulation**	Quercetin; triptolide; glucose-triptolide conjugates	Potential reduction of hypoxia-driven immunosuppression and metastatic signaling	[[128], [129], [130], [131], [132], [133]]
	**Metabolism remodeling (acidification/ROS)**	Curcumin	Potential restoration of immune cell function; Mitigation of acidity-associated immunosuppression; Interference with ROS-driven tumor immune evasion.	[[134], [135], [136], [137], [138], [139], [140], [141], [142]]
**Immunomodulatory activity**	**PD-L1 targeting immunomodulation**	Quercetin; curcumin; berberine; benzosceptrin C	Relief of checkpoint-mediated immune inhibition; Improved T-cell infiltration and cytotoxicity; Increased sensitivity to immunotherapy;	[[102], [103], [104], [105], [106], [107], [108]]
	**Immunosuppressive cells remodeling**	Triptolide; berberine; resveratrol	Partial reversal of the immunosuppressive microenvironment; Remodeling of Treg/MDSC associated suppression	[106], [107], [[109], [110], [111], [112]]
**Bone regeneration/destruction balance**	**Preservation of bone**	Curcumin	Suppression of tumor associated bone defect	[[113], [114], [115], [116], [117], [118], [119], [120], [121], [122], [123], [124]]
	**Immunoregulatory osteogenesis and repair support**	Curcumin; berberine	Coordination of antitumor immunity with osteogenesis, support for bone defect repair.	[[120], [121], [122], [123], [124], [125], [126]]

### 3.1 Natural bioactives regulating immune function

Building on the immune landscape summarized in Section 2.2, this section focuses on how natural bioactives modulate checkpoint signaling and immunosuppressive cells in malignant bone tumors [[97], [98], [99]]. Studies of existing immune checkpoint inhibitors have shown limited benefit and substantial heterogeneity in the overall population of bone and soft tissue sarcomas [100,101]. In this clinical context, natural bioactives should be positioned primarily as immunosensitizing agents rather than as standalone therapies. Their potential clinical application would require rigorous in *vitro* evidence (e.g., enhanced T-cell cytotoxicity and improved antigen presentation) as well as in *vivo* validation (e.g., restoration of immune function in relevant animal models) before translation into clinical settings.

Current research on natural bioactives has largely focused on modulating immune checkpoint expression. Taking quercetin as an example, in *vitro* studies indicate that it can inhibit the JAK2–STAT3–PD-L1 signaling axis, while in *vivo* work has further enhanced its antitumor efficacy by using folate-modified liposomes for delivery [102]. However, a shared limitation of these studies is that endpoints still tend to rely primarily on reduced PD-L1 expression and changes in tumor burden, with insufficient evidence demonstrating whether effector T cells truly recover from exhaustion and whether any post-intervention functional restoration is durable. Importantly, immune functional recovery should not be inferred solely from changes in inhibitory receptor expression; rather, it should be evaluated comprehensively by integrating cellular differentiation states, epigenetic features, and functional readouts [103,104].

Compared with quercetin, a study on curcumin proposed that NF-κB–driven CSN5 can deubiquitinate and stabilize PD-L1, whereas curcumin inhibits CSN5, thereby reducing PD-L1 levels and increasing sensitivity to immunotherapy [105]. Building on this mechanism, an in *vitro* study of berberine showed that it suppresses the deubiquitinase activity of CSN5 and induces PD-L1 degradation via the ubiquitin–proteasome pathway; in *vivo* experiments further observed increased T-cell infiltration into tumors accompanied by reduced activation of myeloid-derived suppressor cells (MDSCs) and Tregs [106,107]. Notably, evidence for berberine is largely derived from non-bone tumor models. Therefore, the immunomodulatory relevance of berberine in osteosarcoma or other malignant bone tumor models should be regarded as provisional until validated in bone tumor models. As discussed in Section 2.2, hypoxia-driven HIF-1α signaling may counteract PD-L1-lowering strategies, which should be considered when interpreting these findings. Nonetheless, this strategy (promoting the degradation of inhibitory immune checkpoint molecules) appears to yield more definitive immunomodulatory outcomes (e.g., reduced Treg activity and enhanced T-cell infiltration). In addition, in *vitro* studies have demonstrated that Benzosceptrin C decreases PD-L1 abundance and enhances T-cell cytotoxicity, and in *vivo* findings indicate that it activates tumor-infiltrating T-cell immunity and exerts antitumor effects [108]. Collectively, these observations suggest that, relative to suppressing the transcription of immune checkpoints, directly accelerating their degradation may represent a more promising translational route for natural bioactives in immunotherapy for malignant bone tumors.

In addition, natural bioactives are frequently explored for their capacity to modulate immune cell differentiation and recruitment, as well as to regulate the secretion of diverse cytokines. An in *vivo* study reported that triptolide reduced tumor burden and decreased the proportion of Tregs in tumor-bearing mice, accompanied by downregulation of IL-10, TGF-β, and VEGF [109], suggesting potential value in immunotherapeutic settings. Studies on resveratrol further indicate that its effects on macrophage polarization are strongly dependent on concentration and clinical context [110]. However, because the functions of TAMs are highly plastic across time and spatial niches, measurements of M1/M2 markers at a single time point are insufficient to infer whether an intervention can elicit sustained antitumor immunity [111]. Overall, the available evidence suggests that natural bioactives tend to act as immunosensitizing approaches in remodeling the IME of malignant bone tumors [112], and that combining them with other immunotherapeutic strategies may yield improved efficacy.

### 3.2 Natural bioactives regulating bone remodeling

Extending the bone-destructive framework outlined in Section 2.2.3, this section discusses whether natural bioactives can simultaneously restrain osteolysis and restore immune-supportive bone repair [97]. Within the IME of malignant bone tumors, enrichment of myeloid cells, immunosuppression, and bone-remodeling signals coexist, providing an important basis for local osteolysis and for impaired repair after treatment. RANKL is a key factor for osteoclasts and is highly expressed in osteolytic bone tumors. It is associated with bone destruction and is considered one of the potential therapeutic targets for malignant bone tumors [113]. Current perspectives suggest that the RANKL–RANK–OPG signaling axis promotes osteoclast differentiation and bone resorption in various pathological environments. It not only plays a role in bone metabolism but also facilitates tumor immune tolerance by modulating the status of immune cells, particularly through interactions between dendritic cells and T cells [114,115]. Accordingly, the RANKL–RANK axis in immunologically oriented bone tumor therapy may influence both antitumor immunity and bone pathology, providing a theoretical basis for coordinated bone regeneration and immune modulation within our proposed “three-dimensional synergy” immunotherapy framework for malignant bone tumors [116,117].

A key question is whether natural bioactives can simultaneously modulate immune activity and promote bone regeneration. This should be supported by both in *vitro* and in *vivo* evidence and should translate into defect repair while limiting pathological bone destruction. Studies have shown that curcumin can directly inhibit osteoclast function by suppressing the RANKL signaling pathway and regulate stem cell differentiation to promote osteogenesis [118,119]. Additionally, curcumin can enhance immune responses in the tumor microenvironment [120,121]. Although current research evidence comes from non-bone tumor models, which may limit the extrapolation of the conclusions, this suggests that curcumin has therapeutic potential in simultaneously modulating bone remodeling and immune activity. In addition, STAT3 represents an intersectional node linking immunosuppression and bone destruction. The IL-6/JAK/STAT3 axis within the tumor microenvironment not only promotes tumor progression but also suppresses antitumor immune responses; thus, inhibiting this pathway may simultaneously relieve immunosuppression and indirectly shift the milieu toward bone regeneration [122]. From immunological perspective, a clinically relevant large-cohort analysis combined with mechanistic work has indicated that intrinsic IL-6 signaling in CD8^+^ T cells is associated with resistance to anti–PD-L1 therapy, implying that IL-6/STAT3 activation compromises effective cytotoxic T lymphocyte (CTL)–mediated tumor control [123]. From bone regeneration, under inflammatory conditions IL-1β and TNF-α can activate the IL-6–STAT3 pathway and induce RANKL expression, thereby promoting osteoclast differentiation [124]. Together, these findings suggest that curcumin may target both the RANKL-osteoclast axis and IL-6/STAT3-mediated immunosuppression. However, its ability to support both new bone formation and sustained immune surveillance in postoperative bone-tumor defects remains unproven.

In addition, several studies suggest that natural bioactives such as berberine may promote bone regeneration while simultaneously exerting immunomodulatory effects [125,126]. These findings further strengthen the evidence base supporting the potential incorporation of natural bioactives into immunotherapeutic strategies for malignant bone tumors.

### 3.3 Natural bioactives modulating physicochemical properties

Based on the physicochemical abnormalities detailed in Section 2.2.1, this section highlights natural bioactives with evidence for targeting hypoxia adaptation, acidosis, and redox imbalance [97,[127], [128], [129]]. Studies have shown that HIF-1α directly binds to the PD-L1 promoter and upregulates PD-L1 expression in MDSCs and tumor cells under hypoxic conditions, thus enhancing myeloid immune suppression and T-cell dysfunction [107]. Additionally, in the osteosarcoma environment, HIF-1α is also associated with metastasis and drug resistance [130]. Based on this evidence, in *vitro* studies have demonstrated that quercetin inhibits HIF-1α accumulation and reduces VEGF output under 1% O_2_ conditions, directly suppressing the initiation of hypoxia-related pathways [131]. In *vitro* studies also suggest that triptolide may attenuate hypoxia adaptation by inhibiting HIF-1 transcriptional activity or downstream target gene expression in various models [132]. To overcome the toxicity and delivery limitations of triptolide, strategies such as glucose–triptolide conjugates have been developed for enhanced tissue selectivity and efficacy under hypoxic conditions, providing evidence for new strategies of combining natural bioactives with bioactive materials in synergistic immunotherapy [133].

In terms of IME acidification, tumor glycolysis leads to the accumulation of lactate and protons, creating a low pH microenvironment. Lactate and acidification have a multifaceted impact on T-cell effector function, dendritic cell antigen presentation, and other immune responses. Lactic acid and acidification not only suppress the function of effector T cells but also promote tumor cell immune evasion by reshaping metabolic pathways and enhancing Treg activity. Modulating the acidic tumor microenvironment is still considered an important strategy to enhance the effectiveness of immunotherapy [[134], [135], [136]]. Recent studies also suggest that acidity can suppress CD8^+^ T-cell function by disrupting the IL-2/mTORC1/c-Myc axis and amino acid metabolism [137]. For natural bioactives, studies indicate that curcumin may reshape tumor metabolic programs and affect molecules related to lactate production and transport, potentially alleviating acidification stress [138].

Regarding ROS, the oxidative stress imbalance in the IME not only promotes tumor adaptation but also induces immune evasion by affecting antigen presentation, inflammasomes, and immune effector cell metabolism [139,140]. In *vitro* studies suggest that curcumin can modulate various ROS-related enzyme systems and alter the oxidative stress state of tumor cells [141,142]. Overall, the effects of natural bioactives on the IME of malignant bone tumors are not limited to a single mode of immune modulation, but instead systematically alter the IME from a "three-dimensional synergy" perspective, integrating physicochemical properties, bone regeneration and destruction balance, and immune regulation. While some evidence extrapolated to malignant bone tumor settings still requires further validation, current research results suggest that natural bioactives hold significant clinical translation potential.

### 3.4 Challenges and limitations in the application of natural bioactives

Natural bioactives have demonstrated notable advantages in the remodeling of the IME of malignant bone tumors and in postoperative bone regeneration. However, substantial challenges remain for their clinical translation. First, low bioavailability and limited tissue specificity remain among the most common reasons for suboptimal therapeutic efficacy. Pharmacokinetic studies of curcumin have shown that the exposure of the free parent compound in systemic circulation and tissues is extremely low, with significant variability observed among different formulations [143,144]. This suggests that the effective dose is largely dependent on formulation strategies rather than on the intrinsic properties of the molecule itself. Similarly, quercetin is constrained by poor solubility and extensive first-pass metabolism, and its pharmacokinetic profile can be markedly altered through formulation approaches such as cocrystal engineering [[145], [146], [147]]. These observations further indicate that the immunomodulatory and osteogenic effects of the same natural bioactives cannot be directly compared across different formulations or delivery carriers.

Second, the physicochemical characteristics of the IME, such as hypoxia, acidosis, and reactive oxygen species (ROS) accumulation, may prevent many findings obtained in *vitro* from being reproduced in *vivo* [97,130]. Consequently, conclusions based solely on in *vitro* experiments often overestimate the translational relevance of therapeutic efficacy. In addition, the pleiotropic and off-target properties commonly observed in natural bioactives represent a double-edged sword. While multi-target activity may simultaneously influence shared signaling pathways involved in immune regulation and bone regeneration, such as STAT3 and NF-κB, it also increases the likelihood of divergent or even counteracting effects across different cellular populations, including tumor cells, T cells, tumor-associated macrophages (TAMs), and osteoblasts or osteoclasts [148,149].

Furthermore, the complex origins of natural bioactives often lead to batch-to-batch variability and challenges in quality control, particularly in compound extracts. Variations in source materials and processing methods may result in fluctuations in chemical composition, which can directly translate into differences in immunological activity and toxicity profiles. Finally, toxicity and the narrow therapeutic window of certain potent molecules also impose significant limitations [150,151]. Triptolide and related diterpenoid compounds derived from *Tripterygium wilfordii* represent a typical example; numerous mechanistic studies consistently report multi-organ toxicities, including hepatotoxicity and nephrotoxicity, which significantly restrict their incorporation into long-term systemic immunotherapeutic regimens [[152], [153], [154]].

These limitations partly explain why the most promising translational strategies in this field are increasingly shifting toward synergistic immunotherapeutic approaches that integrate natural bioactives with biomaterials, such as scaffolds, hydrogels, and injectable composite systems. First, biomaterials can convert the systemic low exposure of natural bioactives into localized high exposure with controllable release profiles [155,156]. By exploiting IME-associated stimuli, including acidic pH, ROS, and enzymatic activity, stimuli-responsive delivery systems can enhance immunomodulatory effects without increasing systemic toxicity. Second, the biomaterials themselves (e.g., ion-releasing systems, bioceramics, and GelMA-based matrices) can provide an osteogenic microenvironment and immunomodulatory cues, thereby further promoting coordinated bone regeneration and immune regulation [157,158]. Notably, in *vivo* studies using postoperative tibial osteosarcoma models have demonstrated that nano-composite hydrogels enabling the co-delivery of Mg^2+^ and anti-PD-L1 antibodies can simultaneously restore CD8^+^ T cell function and enhance bone regeneration [155]. Such findings provide representative high-quality evidence supporting the combined application of natural bioactives and bioactive materials.

## 4 Bioactive material design for the IME of malignant bone tumors

Bioactive materials can play a regulatory role in remodeling the IME of malignant bone tumors through controlled delivery and local microenvironmental modulation. On the one hand, these materials can precisely regulate the physicochemical properties of the tumor microenvironment, such as hypoxia, acidosis, and ROS levels, and influence the expression of immune checkpoints as well as the recruitment, activation, and functional maintenance of immune cells through the controlled release of therapeutic agents or bioactive factors. In doing so, they help improve immune surveillance and enhance antitumor immune responses. On the other hand, when used as carriers or scaffold systems for bone repair, bioactive materials can simultaneously inhibit tumor-associated bone destruction while providing an osteogenic microenvironment and structural support. This dual functionality promotes postoperative bone regeneration and may also contribute to reducing the risk of tumor recurrence (Table 3).

**Table 3:** Bioactive materials for remodeling the IME of malignant bone tumor.

Characteristics		Bioactive materials	Impact on IME	Reference
**Physicochemical Properties**	Redox-active/ion-releasing materials	Magnesium-based biomaterials; MgO-containing materials; calcium–silicate bioceramics	Contributes to correction of adverse physicochemical conditions in the IME and creates a microenvironment for downstream immune activation and osteogenesis.	[[160], [161], [162], [163], [164], [165], [166]]
	Metal-related materials	Metal–polyphenol network systems; metal ion/redox-responsive material systems	Supports microenvironmental reprogramming toward an antitumor state.	[170], [171]
**Immunomodulatory**	Macrophage reprogramming materials	Calcium–silicate bioceramics; degradable magnesium alloys; immunoregulatory inorganic biomaterials	Improve immune surveillance, and establish an immune context favorable for bone regeneration.	[[160], [161], [162], [163], [164]]
	Immune activating materials	Hydrogels and immunoregulatory material systems	Strengthens antitumor immunity, helps reduce recurrence risk, and links immune activation to reparative remodeling in the IME.	[97], [155], [157], [159], [[167], [168], [169]]
**Bone regeneration**	Immune mediated osteogenic materials	Calcium–silicate bioceramics; degradable magnesium alloys	Promotes osteogenic differentiation, vascularized repair, and reconstruction of pathological bone defects after tumor treatment.	[163], [164]
	Temporally responsive materials	Time-sensitive Mg^2+^-related biomaterials and other sequentially acting bioactive systems	Favors coordinated progression from immune remodeling to bone healing and improves the structural repair after tumor resection.	[165], [169]

### 4.1 Bioactive materials for immunomodulation and osteogenesis

Immunotherapy for malignant bone tumors, particularly osteosarcoma, is characterized by dual therapeutic objectives, the establishment of adaptive immune responses to prevent long-term recurrence and the reconstruction of large pathological bone defects. In recent years, a growing consensus has emerged that the central contribution of bioactive materials is no longer limited to serving as drug carriers or defect fillers. Instead, increasing attention has been directed toward leveraging their delivery capabilities and intrinsic bioactivity, enabling immunomodulation within the tumor microenvironment to act as an upstream driving force for bone regeneration [97,157,159].

**Fig. 5:**
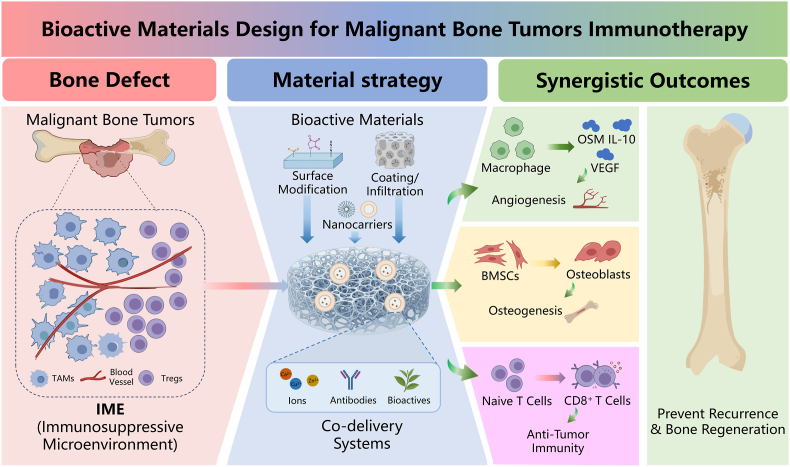
Bioactive materials regulate immune activity and the bone regeneration process. These materials are primarily used for drug loading through surface modification, coating, or infiltration, and in some cases, they achieve the temporally controlled release of loaded agents by incorporating nanocarriers. In this way, they can meet the sequential signaling demands required for both bone defect repair and immunoregulation.

The surface chemistry, microstructure, and ion release characteristics of biomaterials can drive macrophage polarization from a persistently pro-inflammatory state toward a reparative phenotype [[160], [161], [162], [163]]. This transition subsequently promotes osteogenesis and angiogenesis of bone marrow–derived mesenchymal stem cells (BMSCs) through mediators such as oncostatin M (OSM), IL-10, and VEGF. For example, calcium–silicate bioceramics have been shown to enhance the osteogenic differentiation of BMSCs by inducing macrophage-associated signaling and activating the OSM–ERK/JAK pathway [163]. Degradable magnesium alloys, meanwhile, can promote macrophage secretion of IL-10 in *vivo* and drive osteogenesis of periosteum-derived cells through JAK/STAT-related signaling. Notably, inhibition of IL-10 attenuates the bone formation induced by these materials, indicating that their regenerative capacity is indeed immune mediated [164]. In addition, studies on Mg^2+^–TRPM7 signaling highlight that the effects of magnesium ions in both immune regulation and tissue regeneration are dependent on dose and exposure duration. This suggests that the ion release profiles of biomaterials should be aligned with the temporal requirements of adaptive immune establishment against tumors as well as the sequential processes involved in bone defect repair [165]. Importantly, the immunomodulatory activity of such materials often converges on signaling nodes, such as STAT3 and NF-κB, that are also commonly targeted by natural bioactives. However, biomaterial-based strategies offer a more controllable mode of intervention, which may partially compensate for the limitations of natural bioactives, including low bioavailability and insufficient tissue specificity (Fig. 5).

Achieving local immune de-repression together with synchronized osteogenic signaling may represent a feasible biomaterial-based immunotherapeutic strategy for preventing recurrence and reconstructing bone defects in malignant bone tumors. An in *vivo* study demonstrated that a nanocomposite hydrogel capable of sustained co-delivery of Mg^2+^, anti–PD-L1 antibodies, and vismodegib within the resection cavity enhanced the activity of tumor-suppressed CD8^+^ T cells, inhibited postoperative recurrence, and simultaneously promoted bone regeneration [155]. In another example, an MgO-enhanced injectable adhesive hydrogel utilized the intrinsic redox activity of the material together with the sequential action of the hydrogel system to temporally couple immunomodulation with bone regeneration [166]. At the same time, activation of innate immunity also plays an important role in the reconstruction of the IME in malignant bone tumors. Hydrogel microspheres that enable photo/thermal-triggered or sustained delivery of STING agonists have been shown to enhance type I interferon signaling, improve T-cell infiltration, and strengthen antitumor immune responses, providing a potential strategy to convert the “cold” immune phenotype of osteosarcoma into a more immunologically active “hot” state [167,168]. Taken together, immune activation and bone repair are not inherently conflicting processes. Rather, the key to achieving synergy lies in carefully controlling the release kinetics and dosage of the delivered agents [169].

In recent years, metal-related immunosensitization mechanisms have been further extended from metal-induced ferroptosis in tumor cells to the enhancement of tumor immunogenicity. One study proposed a metal–polyphenol network system that amplifies lipid peroxidation to induce ferroptosis and generates a cascade amplification effect when combined with immunotherapy, suggesting that ferroptosis may serve as an immunosensitizing strategy within biomaterial-based therapeutic platforms [170]. Another study indicated that a tunable synergistic relationship exists among metal ions, oxidative stress, and immune responses, providing a theoretical basis for incorporating metal redox reactions into immunotherapeutic strategies based on bioactive materials [171]. Overall, the mechanisms of bioactive materials and natural bioactive compounds in the context of malignant bone tumors partially overlap. This implies that a more rational strategy is not simply to reinforce the same mechanisms redundantly, but rather to use biomaterials to address challenges such as temporally controlled drug release and tissue specificity, while natural molecules contribute complementary multi-target immunomodulatory effects. Such a coordinated approach may ultimately enable synergistic immunotherapy that both suppresses tumor recurrence and promotes bone regeneration.

### 4.2 Bioactive materials as delivery platforms for natural bioactives

**Fig. 6:**
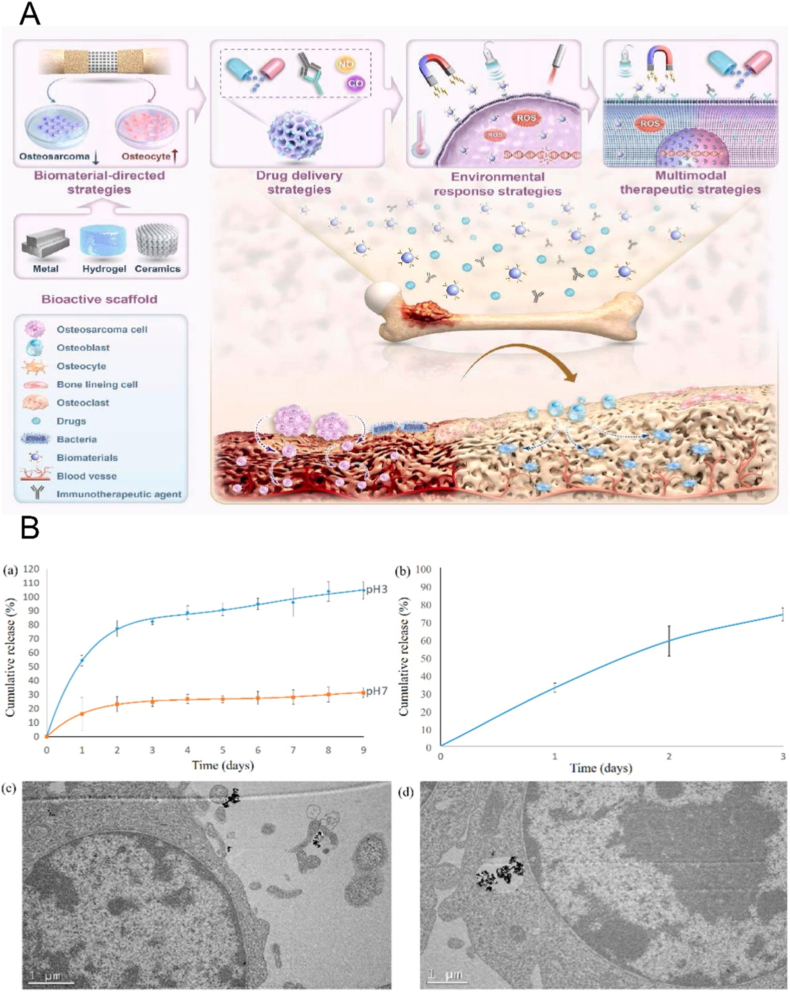
(A) Bioactive materials commonly used for bone defect repair should not only possess stimulus-responsive release capabilities under specific microenvironmental conditions, but also regulate the balance between osteogenesis and osteoclastogenesis [174]. (B) Natural bioactives can achieve sustained release under neutral conditions, while exhibiting accelerated release in acidic environments in an acid-responsive manner [175].

As summarized in Section 3.4, the poor solubility, rapid metabolism, and limited tissue specificity of many natural bioactives have motivated their incorporation into localized biomaterial-based delivery systems [[172], [173], [174]] (Fig. 6).

A commonly employed strategy involves loading natural bioactives through interactions between material surface chemical groups and the natural bioactives. For instance, phosphate and hydroxyl groups on the surfaces of hydroxyapatite or calcium phosphate ceramics can form hydrogen bonding or electrostatic interactions with polyphenolic natural molecules, enabling stable adsorption. Studies have shown that when curcumin is loaded onto hydrophobic surface–modified hydroxyapatite, the drug loading capacity can reach approximately 17.6 wt%, exhibiting an initial burst release followed by a more stable release profile [175]. Similarly, berberine can be incorporated through interactions with surface functional groups of porous calcium phosphate ceramics, achieving a composite content of approximately 0.6 wt%. In vitro experiments have demonstrated sustained release for about 9–10 days, along with the promotion of bone-related gene expression [176]. This surface functionalization–based loading approach is relatively straightforward and well suited for rapid functionalization of bone substitute materials. Moreover, it allows the maintenance of relatively stable local drug concentrations at the implantation site.

Another approach involves direct coating or infiltration-based loading, which is commonly applied in 3D-printed bone scaffolds or porous ceramic materials. In this method, natural molecules are distributed relatively uniformly within the porous structure by directly dripping or soaking the scaffold in a solution containing the bioactive compound. For example, curcumin can be effectively incorporated into MgO-doped β-TCP 3D-printed scaffolds through infiltration into their porous structure. In terms of release behavior, the system shows a cumulative release of about 17% over 30 days under physiological conditions (pH 7.4), whereas the release proportion increases to approximately 23% under acidic conditions (pH 5.0) [177]. This suggests that tumor- or inflammation-associated acidic microenvironments may promote drug release. Such pH-dependent release behavior provides a potential environment-responsive mechanism for postoperative local therapy, whereby drug release is enhanced in tumor-related microenvironments while remaining relatively limited in normal tissues.

To further improve loading efficiency and achieve prolonged release control, increasing attention has been directed toward multilevel delivery architectures. In such systems, natural molecules are first encapsulated within nanocarriers, such as liposomes, solid lipid nanoparticles, or polymeric micelles, and are subsequently incorporated into hydrogels or porous scaffolds to form composite delivery platforms. For example, when resveratrol is loaded into solid lipid nanoparticles and embedded within a GelMA hydrogel, the system exhibits an initial release of approximately 14% within the first 0.5 day, followed by a sustained release phase, reaching a cumulative release of about 75% over 28 days [178]. This approach significantly prolongs the drug release period. Similarly, when quercetin is loaded into mesoporous bioactive glass (MBG), the drug loading capacity can reach 320.25 mg/g with an encapsulation efficiency of approximately 8.54%. The system shows an initial burst release followed by sustained release for about 21 days, while also modulating macrophage-associated immune responses and promoting bone regeneration [179]. In addition, polymer–calcium phosphate composite scaffolds can enable the co-delivery of multiple natural molecules. For instance, the release proportions of curcumin, resveratrol, and vitamin D_3_ under acidic conditions are approximately 64%, 100%, and 80%, respectively, substantially higher than those observed under neutral conditions, thereby enabling selective drug release in tumor-associated microenvironments [180].

Overall, bioactive materials improve the loading and release control of natural bioactives through surface functionalization, direct coating, and multilevel nanodelivery. Compared with systemic administration, these systems can create a stable local drug reservoir in the resection cavity and help overcome poor bioavailability and limited targeting. Future research should focus on improving loading efficiency while optimizing release kinetics, and integrating stimulus-responsive materials or immune-targeting strategies to achieve more precise and controllable immunotherapeutic interventions.

### 4.3 Challenges in clinical translation of bioactive materials

Despite the significant potential of bioactive materials in modulating the IME of malignant bone tumors and promoting bone regeneration, their clinical translation faces several critical challenges. First, the toxicity and long-term biological effects of these materials require systematic evaluation. Many functional materials rely on metal ion release (e.g., Mg^2+^, Fe^2+^, Zn^2+^) or nanoscale structures to exert antitumor and osteogenic effects, yet excessive ion release or long-term accumulation may induce oxidative stress, cytotoxicity, and tissue damage, and could pose accumulation risks in organs such as the liver and kidneys [174]. Second, photothermal or photodynamic therapy strategies, while enhancing local antitumor efficacy, carry potential risks of thermal injury to surrounding healthy tissue and interference with bone regeneration, particularly because the long-term degradation and clearance mechanisms of some photothermal nanomaterials remain unclear in *vivo* [181]. Third, significant translational barriers persist from experimental research to clinical application, including scalable material fabrication, batch-to-batch consistency, long-term safety evaluation, and reproducibility of therapeutic efficacy in complex clinical environments. Finally, while bioactive materials can enhance antitumor immunity through modulation of macrophage polarization or activation of pathways such as STING, prolonged immune stimulation may lead to chronic inflammation or immune imbalance. Therefore, precise control between immune activation and tissue repair is essential.

Future research should focus on the design of biodegradable materials, precise delivery strategies, and comprehensive long-term safety assessments to facilitate the clinical translation of bioactive materials for the treatment of malignant bone tumors.

## 5 Synergistic strategies of bioactive materials and natural bioactives for immunotherapy of malignant bone tumors

Building on the translational limitations outlined in Section 3.4 and the delivery strategies described in Section 4.2, this section discusses how combined systems generate structural and functional synergy [182]. Recent research has increasingly focused on integrating natural bioactives with bioactive materials as localized delivery platforms, enabling controlled loading and sustained release to improve site-specific bioavailability while reducing systemic side effects. In the context of tumor-induced bone defects, such biomaterial systems can simultaneously provide structural support and serve as functional carriers capable of modulating the tumor microenvironment, thereby creating new opportunities for the coordinated management of tumor control and bone regeneration.

Emerging studies have also demonstrated that certain bioactive materials themselves possess intrinsic immunomodulatory properties. For instance, ruthenium-based nanoreactors can catalytically oxidize glutathione to glutathione disulfide, inducing tumor cell death while promoting macrophage differentiation and restoring immune surveillance [183]. Similarly, cationic platinum prodrug nanoparticles derived from alendronate can activate the STING signaling pathway, effectively reversing the immunosuppressive microenvironment in osteosarcoma [184]. In another example, copper-depositing nanoparticles incorporating the natural compound celastrol induce immunogenic tumor cell death through copper-dependent mechanisms [185]. These findings indicate that bioactive materials and natural bioactive compounds share partially overlapping mechanisms while also exhibiting complementary immunomodulatory functions in remodeling the tumor immune microenvironment.

**Fig. 7:**
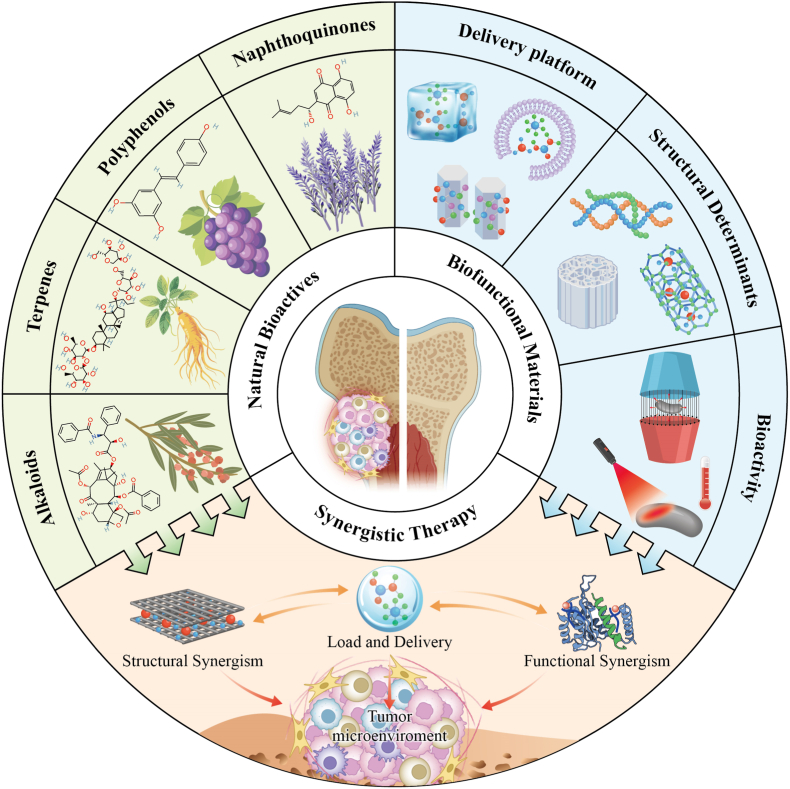
Various natural bioactives, when loaded onto bioactive materials, can enhance tissue specificity while reducing first-pass metabolism and adverse effects. Meanwhile, the structural features of bioactive materials, such as surface topology, size, and morphology, can generate synergistic immunomodulatory effects with natural bioactives. In addition, their functional properties, including photothermal and magnetothermal effects, can further act synergistically with these compounds in promoting bone regeneration and regulating physicochemical properties, thereby collectively contributing to the remodeling of the immune microenvironment of malignant bone tumors.

Building on this understanding, the combination of natural bioactives with bioactive materials can establish a therapeutic strategy characterized by structural synergy and functional synergy (Table 4). Structurally, biomaterials provide scaffolds or localized delivery systems that enable stable loading and sustained release of natural compounds, thereby enhancing their accumulation at tumor sites. Functionally, the intrinsic effects of biomaterials, such as ion release, photothermal activity, or catalytic reactions, can cooperate with the immunomodulatory actions of natural bioactives to regulate immune cell behavior and inflammatory signaling. Together, these synergistic interactions may ultimately enable a “three-axis synergy” therapeutic outcome, simultaneously modulating the physicochemical properties of the tumor microenvironment, enhancing immune activity, and promoting bone regeneration. Such an integrated strategy offers a promising direction for achieving effective tumor suppression while facilitating skeletal repair in malignant bone tumors (Fig. 7).

**Table 4:** Collaborative effects of structural and functional synergy modes.

Mode	Pattern	Immune regulatory	Bone regeneration	Physicochemical remodel	Ref.
**Structural synergy**	Surface loading and direct scaffold loading	Supports localized and sustained exposure to natural bioactives, helping maintain immunoregulatory activity in the IME.	Combines local delivery with scaffold support and osteoconductive matrices for defect repair.	Improves local retention and release control; can be aligned with acidic IME conditions.	[[186], [187], [188], [189], [190], [191]]
	Multilevel and pH responsive delivery materials	Converts the release of compounds such as curcumin into a controllable local delivery process; ICD-related immune activation and immune cell recruitment.	Provides a delivery scheme that can be combined with bone-repair scaffolds.	Uses pH-responsive release and staged delivery to match IME conditions.	[[186], [187], [188]]
	Morphology and biomimetic structures	Certain material morphologies can enhance antigen release, uptake, DC maturation, and DAMP-associated immune initiation.	Biomimetic or cancellous bone-like structures provide mechanical support for bone repair.	Supports local immune activation within the IME.	[[193], [194], [195]]
	Membrane-coated and oxygen-releasing structures	Membrane-coated carriers improve lesion targeting and in vivo stability; oxygen-releasing components help reduce hypoxia-associated immunosuppression.	Provides a more favorable structural basis for repair by improving local transport conditions.	Addresses hypoxia and oxygen-diffusion limitations in scaffolds.	[[196], [197], [198], [199], [200], [201]]
**Functional synergy**	Photothermal therapeutic platforms	Material-generated thermal effects promote tumor cell ablation, antigen exposure, and immune activation; these effects can be further integrated with natural bioactives.	Photothermal scaffold systems can retain bone-repair support while reducing tumor burden.	Acts on the local IME through heat-triggered tumor damage.	[169], [202], [203]
	Piezoelectric bioactive materials	Ultrasound-driven electrical signaling is reported to promote macrophage polarization, DC maturation, and DAMP release.	Bioactive glass and Sr-related components support BMSC osteogenic differentiation and accelerate repair of bone defects.	Generation of ROS and regulation of acidic microenvironment are controlled by electrical signal transduction.	[[204], [205], [206], [207]]
	Polysaccharide-metal hydrogel	Controlled Cu^2+^ release together with the polysaccharide matrix is reported to promote M1 polarization, DC maturation, and increased CD8^+^ T-cell infiltration.	Platform has been reported to support osteogenic differentiation while suppressing osteosarcoma growth.	Remodels the local IME through sustained metal-ion release and cuproptosis.	[208], [209]
	Sonodynamic and metabolic modulation platforms	Ultrasound contributes to tumor-cell killing and can be combined with natural bioactives for immunotherapy-oriented treatment.	Bone-regeneration support usually depends on additional scaffold or bioactive-material modules combined with the sonodynamic system.	Reported to suppress glycolysis-related acid accumulation and reduce HIF-1α and lactate levels.	[[210], [211], [212], [213]]

### 5.1 Structural synergy with natural bioactives

When the combined use of bioactive materials and natural bioactives is interpreted merely as the material-based delivery of natural products, the underlying synergistic mechanisms are often reduced to simple pharmacokinetic improvements, such as enhanced solubility, prolonged retention, or controlled burst release. However, in the postoperative setting of malignant bone tumors, the true upper limit of therapeutic efficacy is determined by whether the immunomodulation, bone regeneration, and physicochemical microenvironment remodeling can be simultaneously driven by a single system in a sustained and verifiable manner. In this context, the essence of a structural synergy strategy lies in using material architecture, morphology, porosity/interconnectivity, and interfacial chemical properties to amplify the molecular pharmacological effects of natural bioactives, thereby providing a stable foundation for this three-axis coordination.

ZIF-8, a zinc-based metal-organic framework (MOF), has been employed for pH-responsive release in the acidic tumor microenvironment to address the poor solubility, rapid metabolism, and requirement for high local exposure associated with natural bioactives such as curcumin. The significance of such systems lies not only in precise release, but also in their ability to convert the immunomodulatory effects mediated by natural products into a controllable material-based modality [186,187]. Curcumin-loaded ZIF-8 can induce autophagy regulation and trigger immunogenic cell death (ICD) at the tumor site, thereby promoting the transformation of poorly infiltrated “cold tumors” into “hot tumors” with enhanced immune cell recruitment. In addition, the loading capacity of ZIF-8 enables the co-delivery of immunoadjuvants, further amplifying synergistic immune effects.

A similar rationale is also reflected in the structural synergy achieved through the co-delivery of ions and natural products. When a system simultaneously delivers Ca^2+^ and curcumin, the carrier can, on the one hand, exploit curcumin to inhibit calcium release from endoplasmic reticulum stores, thereby enhancing tumor cell apoptosis and promoting the polarization of TAMs toward the M1 phenotype. On the other hand, the release of Ca^2+^ and related ionic species can promote the osteogenic differentiation of locally resident stem cells, thus integrating antitumor immunity and bone defect regeneration within a unified immunotherapeutic framework [186,188]. Therefore, the evaluation of structural synergy should not remain limited to higher drug loading or more stable release profiles. Rather, it should focus on whether the delivery architecture drives the immunomodulatory axis and the bone regeneration axis into a genuinely cooperative range. For example, it should be determined whether the immunoregulatory activity is temporally aligned with the immune-mediated bone regeneration processes discussed earlier.

Natural bioactives such as polyphenols, flavonoids, and catechol-containing compounds possess abundant phenolic hydroxyl groups and aromatic ring structures, enabling them to form stable interfaces through covalent grafting, metal–phenolic networks (MPNs), hydrogen bonding, and π–π stacking. In this way, the functions of natural bioactives can be integrated into bioactive materials as durable functional interfaces, while on-demand release can be achieved in response to IME cues such as acidification and ROS accumulation. Bifunctional agents such as APTES can secure natural bioactives onto inorganic material surfaces through surface-adhesive strategies, allowing stable immobilization and sustained release. Because this approach proceeds under mild reaction conditions and avoids strong acids or bases as well as high temperature and pressure, it is more favorable for preserving the bioactivity of natural bioactives, while sustained release helps maintain effective concentrations and biosafety; for example, the release proportion in simulated body fluid (SBF) remains very low over 48 h^189^. Covalent grafting of EGCG onto the surface of nanohydroxyapatite via amide bonds can markedly improve stability and prevent burst release, while also achieving synergistic antitumor and osteogenic effects in the postoperative bone tumor setting [190]. Owing to their universal adhesiveness, pH responsiveness, tunable mechanical properties, and multifunctional synergy, metal–phenolic networks (MPNs) are now widely regarded as promising interfacial platforms for the simultaneous regulation of inflammation, vascularization, and osteogenic differentiation [191,192].

Material morphology can directly shape the immune microenvironment. For example, rod-shaped nano-hydroxyapatite (approximately 500 nm of length) enhances antigen release, dendritic-cell maturation, and DAMP release. Thus, material structure is not merely a passive carrier feature; it can actively determine whether immune activation is initiated [193]. On this basis, the incorporation of natural products, such as polyphenols, terpenoids, and alkaloids that regulate pathways including STAT3, NF-κB, and PD-1/PD-L1, is more consistent with the therapeutic strategy of “three-axis synergy.” In such a framework, the structure of the bioactive material enhances immune responsiveness, while the natural bioactive components further potentiate the immune reaction, ultimately facilitating the establishment of durable antitumor immunity and bone regeneration within the IME. In addition, twisted nanofibers formed through the self-assembly of L-/D-leucine-enriched phosphopeptides have been shown to selectively kill osteosarcoma cells within the nucleus [194], whereas 3D-printed scaffolds that mimic cancellous bone architecture can suppress MG-63 cell adhesion and growth while simultaneously providing mechanical support, thereby offering a structural platform for the tissue-specific delivery of natural products to pathological bone defects [195]. Taken together, these studies indicate that structural synergy strategies do more than improve drug delivery; they also provide a new approach for remodeling the IME of malignant bone tumors through the coordinated regulation of immune activity and bone regeneration.

Cell membrane-coated nanoparticles not only improve tumor targeting and in *vivo* stability, but their preparation methods and mechanisms of action have also evolved into a relatively well-established theoretical framework [196]. In the osteosarcomas [197], tumor cell membrane-coated systems can deliver natural bioactive compounds to the lesion more stably and enable their combination with immunotherapy or chemotherapy. For example, a tumor cell membrane-coated hollow MnO_2_ carrier loaded with ginsenoside Rh2 has been applied in MRI-guided chemodynamic therapy (CDT) combination treatment, providing an illustrative example of the synergistic integration of membrane-structured materials and natural bioactive components in immunotherapy [198].

Moreover, with respect to regulating the physicochemical properties of the IME in malignant bone tumors, oxygen-releasing components such as CaO_2_ and CPO can provide sustained oxygen supply through hydrolysis, thereby alleviating cellular dysfunction and hypoxia caused by insufficient oxygen diffusion within scaffolds [[199], [200], [201]]. Incorporating such oxygen-generating structures into postoperative bone tumor material systems can, at the structural level, directly counteract hypoxia-induced immunosuppression and the risk of repair failure, thus providing a more favorable physicochemical foundation for the immunomodulatory and osteogenic effects of natural products.

### 5.2 Functional synergy with natural bioactives

Materials can exert immunotherapeutic effects within the IME of malignant bone tumors through the conversion of various forms of energy, including thermal, acoustic, magnetic, and electrical energy. In contrast to structural synergy strategies, functional synergy strategies operate by actively killing malignant bone tumor cells to enhance antigen presentation efficiency. At the same time, they enable stimulus-responsive drug release, particularly of natural bioactives, to exert immunomodulatory effects, promote bone regeneration, and restore the physicochemical balance of the IME.

In the osteosarcomas, multifunctional scaffolds based on photothermal effects can simultaneously achieve osteosarcoma cell ablation and bone tissue regeneration [202]. At the same time, existing studies have shown that, within the IME of malignant bone tumors, mild photothermal treatment can accelerate regeneration and improve the local microenvironment [203]. A recent study further demonstrated that a biodegradable bone scaffold platform can integrate local photothermal–chemotherapeutic treatment with immunotherapeutic synergy while also supporting bone repair. In this system, a Pt (IV) prodrug is activated in a high-GSH environment and subsequently triggers STING signaling, thereby enabling not only drug delivery but also activation of the innate immune system [169]. Collectively, these findings suggest that the active tumor-killing effects generated by materials can do more than simply eradicate tumor cells; they can also be integrated with immunoregulatory modulation and the promotion of bone regeneration.

A unique advantage of piezoelectric materials lies in their ability to convert endogenous mechanical stimulation or externally applied ultrasound into quantifiable electrical signals, thereby initiating immunoregulatory processes. Ultrasound-driven piezoelectric discharge can generate localized electrical stimulation and induce macrophage polarization toward a pro-inflammatory phenotype, with the underlying mechanism involving Ca^2+^ influx and activation of the Ca^2+^–CAMK2A–NF-κB pathway [204]. Recent studies have further demonstrated that ultrasound-activated piezoelectric nanoparticles can modulate macrophage polarization through electrical signaling, providing additional experimental evidence for bioelectric–immune regulation [205].

In the context of tumors, ultrasound-activated piezoelectric nanostructures not only directly inhibit tumor growth, but also remodel the tumor microenvironment through the Ca^2+^/NF-κB axis [206]. A recent study on the synergistic application of natural bioactive compounds and piezoelectric bioactive materials showed that ultrasound-triggered ROS, acting as an “external input” signal, can induce tumor cell apoptosis. Meanwhile, the acidic IME of osteosarcoma enhances the stability of EGCG and promotes the reversal of DNA demethylation, serving as an “internal input” signal that strengthens pyroptosis-related processes. Together, the externally induced ROS and the internally mediated epigenetic regulation form an “external piezoelectric–internal epigenetic” gating mechanism that triggers PANoptosis, completely disrupts cellular barriers, and promotes the release of DAMPs, thereby facilitating dendritic cell maturation and enhancing antitumor immune responses. Finally, bioactive glass (BG) scaffolds and the bioactivity of Sr promote the osteogenic differentiation of BMSCs, accelerating bone defect repair and enabling bone regeneration [207]. This study provides a valuable paradigm for the development of immunotherapeutic strategies centered on the integrated goals of immunoregulation, bone regeneration, and physicochemical remodeling of the microenvironment.

The natural *Tremella fuciformis* polysaccharide (TFP) coordinates copper ions via carboxyl and hydroxyl groups to form a hydrogel-like therapeutic platform [208]. This system demonstrates sustained and controlled metal ion release, suppressing K7M2 osteosarcoma cell proliferation while promoting osteogenic differentiation and inducing macrophage M1 polarization. These effects collectively inhibit tumor growth in vivo in tumor-bearing mice. Furthermore, released copper ions (Cu^2+^) potentiate tumor cell cuproptosis and enhance dendritic cell maturation, thereby increasing CD8^+^ T-cell infiltration within tumor tissues [209].

The core mechanism of sonodynamic therapy (SDT) lies in the activation of sonosensitizers by low-intensity ultrasound to generate reactive oxygen species (ROS) and induce tumor cell death. In recent years, the design strategies of nanosonosensitizers have been extensively investigated [[210], [211], [212]]. One study combined SDT with glycolysis inhibition, using SHK@Mn-TiO_2_ as an SDT platform that simultaneously downregulated the HK-2/PKM2 pathway to suppress glycolysis, thereby markedly reducing HIF-1α and lactate levels and reversing the hypoxic microenvironment [213]. This illustrates the synergistic interplay between natural bioactive compounds and bioactive materials in metabolic regulation and physicochemical remodeling of the microenvironment.

The synergistic application of natural bioactives and bioactive materials can achieve “three-axis synergy” through both structural and functional integration. In this framework, the material component provides the foundation for tissue specificity, interfacial interactions, and the remodeling of the physicochemical microenvironment, including hypoxia, acidification, and ROS imbalance, while the natural bioactives exert multitarget immunomodulatory effects. Together, these two components initiate antitumor immunity and couple it with ion-mediated or scaffold-mediated osteogenic signaling, ultimately enabling the simultaneous realization of immune remodeling, promotion of bone regeneration, and regulation of the physicochemical properties of the IME.

## 6 Discussion and perspectives

### 6.1 Advantages of the synergistic application of natural bioactives and bioactive materials

This review delineates the multifaceted roles of natural bioactive compounds in countering immune evasion by malignant bone tumor cells. These compounds disrupt the immunosuppressive microenvironment through multimodal mechanisms, including the modulation of cytokine networks (e.g., downregulation of TGF-β/IL-10), inhibition of immune checkpoint molecules (PD-L1/CTLA-4), and reprogramming of immunosuppressive cells (regulatory T cells, tumor-associated macrophages) [[214], [215], [216]]. Concurrently, the review summarizes how bioactive materials exert synergistic effects by compensating for the limitations of natural bioactive components. By loading these compounds, bioactive materials enhance tissue specificity through localized injection and targeted modifications, thereby intelligently mitigating the side effects associated with the broad-spectrum immunomodulatory actions of the natural agents. Furthermore, they maintain effective plasma concentrations and ensure medium-to long-term therapeutic efficacy via controlled and sustained release. The release of metal ions and combination with physical therapies such as photothermal therapy (PTT) and sonodynamic therapy (SDT) can further assist the immunomodulatory functions of the natural compounds. While synergistically modulating immunity, certain scaffold materials also provide biomechanical support for pathological bone defects caused by malignant bone tumors and promote bone repair and osseointegration.

For delivery systems, carriers that exhibit complementary functions with the loaded natural bioactives may demonstrate enhanced immunomodulatory activity. For example, functionally modified nano-calcium carbonate or nano-hydroxyapatite particles, as mentioned above, degrade in the hypoxic and slightly acidic microenvironment of malignant bone tumors and help moderate local pH levels. This effect synergizes with the ability of natural bioactives such as shikonin and curcumin to modulate the hypoxic microenvironment [217,218], potentially leading to stronger therapeutic outcomes. Similarly, metal ions (e.g., manganese) released from mesoporous bioactive glass materials inherently exert immunomodulatory effects. When loaded with ginsenoside Rh2 [219], such systems can collectively influence the establishment of adaptive immunity by regulating T-cell activity and proliferation, as a synergistic strategy that may broaden the efficacy of immunotherapy. Furthermore, natural bioactives (e.g., quercetin) encapsulated in macrophage or tumor cell membranes, or modified with folic acid, can significantly improve tissue specificity and bioavailability [220,221]. Therefore, selecting appropriate carriers based on the specific properties of natural bioactives represents a critical direction for future research.

### 6.2 Clinical performance of synergistic therapeutic strategies in clinical trials

Building on the preliminary experimental validation of synergistic immunomodulatory effects, emerging clinical evidence has begun to support a feasible translational pathway that integrates natural bioactives with bioactive material-based strategies. A therapeutic intervention study of high-dose resveratrol in patients with low-grade gastrointestinal neuroendocrine tumors demonstrated its tolerability and explored mechanisms associated with the Notch-1 signaling pathway (NCT01476592), thereby providing clinical support for dosing regimens aimed at molecular pathway modulation through sustained administration. Meanwhile, a comparative multidose pharmacokinetic study focused specifically on the systemic and tissue exposure of curcumin by quantitatively measuring its concentrations in blood and rectal tissue under different formulation conditions (NCT01330810), thereby providing a useful reference framework for formulation selection.

Notably, several registered studies have moved beyond conventional oral supplementation and toward material-based formulations that more directly reflect the concept of synergistic application. A representative example is the clinical trial evaluating liposomal curcumin in combination with radiotherapy and temozolomide for newly diagnosed high-grade glioma (NCT05768919). This study was designed to examine whether carrier-enabled improvements in systemic exposure and tumor-specific accessibility can support combination treatment in a defined clinical setting. Similarly, a clinical trial investigating plant exosome-mediated delivery of curcumin to normal colon tissue and colon tumors (NCT01294072) is conceptually highly consistent with the co-design of natural bioactive components and biomaterials. In this context, the exosome-like vesicular carrier is not merely a transport vehicle; rather, its purpose is to address the longstanding translational limitation of free curcumin associated with poor bioavailability and to enhance tissue deposition at sites where microenvironmental remodeling is required. Complementing these efforts, a study using nanomicellar curcumin was designed to assess biomarkers of oxidative stress and inflammatory signaling, including NF-κB in peripheral blood mononuclear cells, in individuals with metabolic syndrome following intervention (NCT03514667). Together, these studies suggest that, in clinical research, formulations combining nanocarriers with natural bioactive compounds are evaluated not only through clinical outcomes, but also through blood-based readouts that reflect underlying disease mechanisms.

Beyond curcumin and resveratrol, quercetin has also shown early translational potential. A controlled study in postmenopausal women, designed around bone turnover markers and inflammatory cytokines (NCT05371340), provides a clinically relevant link between immune-related biomarkers and bone metabolism. In oncology-related translational research, another study investigated the anticancer activity of quercetin encapsulated in PLGA-PEG nanoparticles in models of tongue squamous cell carcinoma (NCT05456022), reflecting a shift in clinical research from asking whether the compound itself is effective to evaluating whether its therapeutic efficacy can be enhanced through synergistic application with bioactive material-based strategies.

**Fig. 8:**
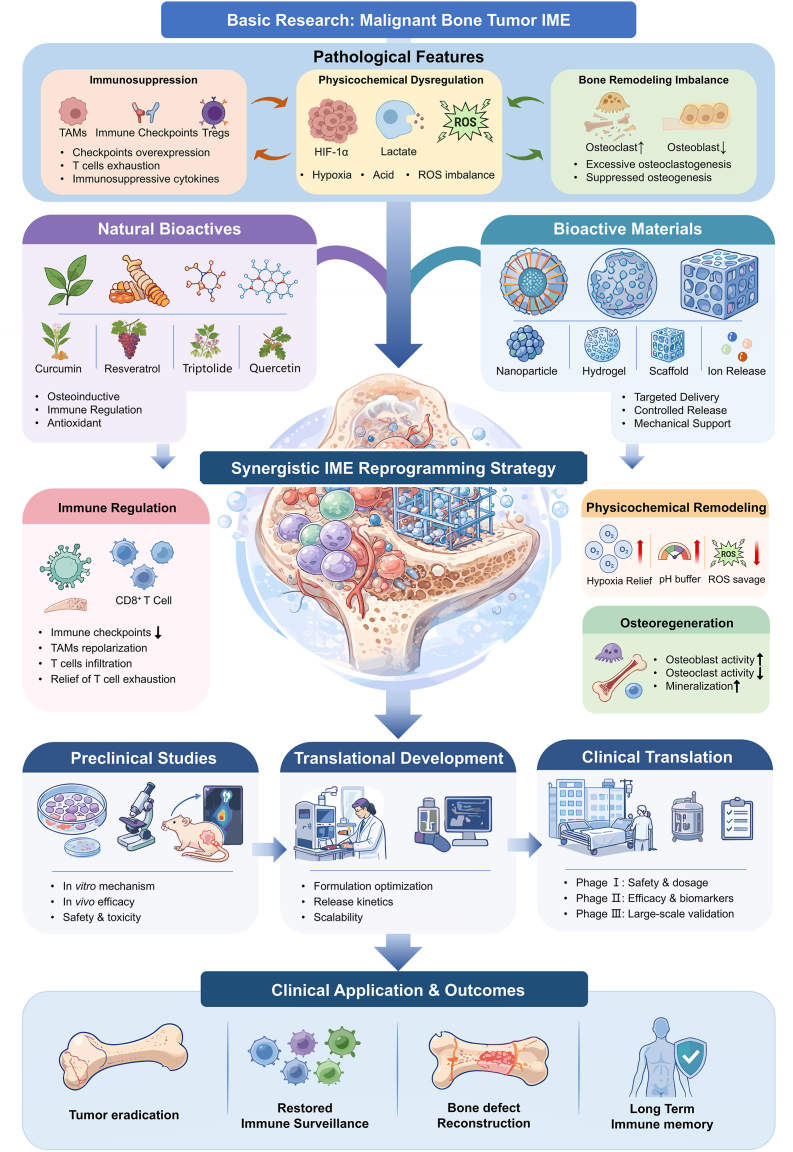
A possible route for the clinical translation of this synergistic strategy. First, basic research should be conducted to investigate the mechanisms underlying the “three-axis synergy” between natural bioactives and bioactive materials. Next, the two should be matched according to requirements for delivery efficiency and regulatory activity. This should be followed by in vitro and in vivo evaluations of safety, biotoxicity, and delivery efficiency. After completion of preclinical studies, the process can proceed to large-scale production steps, including structural optimization and stability testing. Finally, phase I, II, and III clinical trials should be conducted to determine therapeutic efficacy.

Overall, the current clinical evidence remains insufficient to support the combined use of natural bioactives and bioactive material-based strategies as a superior immunotherapeutic approach for routine clinical application (Table 5). Nevertheless, the available clinical findings do provide an initial direction for the future development of this “three-axis synergy” therapeutic framework. First, further studies are needed to clarify differences in metabolism and tissue accumulation among different batches and formulations of natural bioactives. Second, material-based strategies should be further optimized to achieve, as far as possible, quantifiable and comparable improvements in therapeutic efficacy, together with corresponding changes in relevant blood-based biomarkers, so that clinical trial outcomes can more closely align with the intended “three-axis synergy” immunotherapeutic strategy (Fig. 8).

**Table 5:** Clinical trials.

Study/NCT	Natural bioactives and strategy	Tumor type/clinical context	Phase and current status	Key lessons
NCT01476592	High-dose resveratrol monotherapy; sustained administration aimed at molecular pathway modulation through Notch-1 signaling.	Low-grade gastrointestinal neuroendocrine tumors (solid tumor).	Completed; early biological/therapeutic intervention study.	Natural bioactives can enter clinical testing as mechanism-oriented interventions. Future studies should better link pathway modulation to measurable antitumor benefit.
NCT01330810	Curcumin under different formulation conditions; comparative multidose pharmacokinetic study focused on systemic and tissue exposure.	Colorectal/rectal tissue exposure study in a solid-tumor-related setting.	Completed; pharmacokinetic study.	Metabolism, systemic exposure, and tissue accumulation of different curcumin formulations need to be clarified and standardized.
NCT05768919	Liposomal curcumin in combination with radiotherapy and temozolomide; carrier-enabled strategy to improve exposure and tumor accessibility.	Newly diagnosed high-grade glioma (solid tumor).	Recruiting; interventional combination study.	This is one of the clearest examples of a biomaterial-enabled natural bioactives strategy moving into oncology. The major lesson is that delivery technology is being used not merely as a vehicle, but as a way to overcome the longstanding translational limitation of poor bioavailability.
NCT01294072	Plant exosome-mediated delivery of curcumin; exosome-like vesicular carrier designed to enhance tissue deposition.	Normal colon tissue and colon tumors (solid tumor context).	Unknown status; last known status recruiting.	Window-of-opportunity and tissue-distribution studies are highly valuable for natural bioactives. The key lesson is that improved local delivery and tissue deposition are central to translation, especially when free compounds have poor bioavailability.
**NCT05456022∗**	Quercetin versus quercetin encapsulated in PLGA-PEG nanoparticles; oncology-related translational comparison.	Tongue squamous cell carcinoma model/oncology-related translational research.	Unknown status; registered as Phase 2.	This study provides a possible effective transformation path for the synergistic application strategy of natural bioactives and bioactive materials.

### 6.3 Challenges and perspectives

Overall, the clinical translation of immunotherapeutic strategies jointly constructed from natural bioactives and bioactive materials still faces several key challenges.

First, there is still a lack of precise matching between material release behavior and the pharmacokinetic characteristics of natural bioactives. Many natural bioactives suffer from low bioavailability, rapid metabolism, and insufficient tissue accumulation. Future efforts should optimize material design by integrating the pharmacokinetics, tissue distribution, and microenvironment-responsive mechanisms of natural bioactives, so as to achieve more rational temporal release profiles and tissue-specific delivery.

Second, the evaluation system for long-term immune effects remains inadequate. Most current studies mainly focus on short-term tumor inhibition or tissue repair outcomes, while lacking systematic assessment of long-term immune memory and immune homeostasis maintenance. Subsequent studies should strengthen the standardized characterization of adaptive immunity in order to improve comparability across studies and enhance mechanistic interpretation.

Third, clinical translation still faces the dual barriers of safety and industrialization. Long-term immune regulation may lead to risks such as chronic inflammation, abnormal bone remodeling, and systemic toxicity, while multicomponent material systems also face problems including poor batch-to-batch stability, complex preparation procedures, and difficulties in large-scale production. Therefore, long-term toxicological evaluation, safety validation, and the establishment of scalable and standardized manufacturing processes need to be advanced in parallel during the early stages of research.

Fourth, future clinical translation pathways should place greater emphasis on stepwise validation of synergy. At the present stage, a more feasible direction is to begin with systems that have relatively simple components and well-defined mechanisms, and to prioritize validation of their improvements in delivery efficiency, immune-related biomarkers, and bone repair indices, thereby gradually advancing therapeutic outcomes toward the “three-axis synergy” goal of immune regulation, tissue repair, and physicochemical property remodeling.

In summary, major translational challenges remain, but the field now has a clearer developmental framework. Combining natural bioactives with bioactive materials improves local retention and tissue specificity while enhancing IME remodeling and sustained antitumor surveillance. This integrated strategy may offer a more coordinated approach to immune regulation, bone repair, and physicochemical remodeling.

## CRediT authorship contribution statement

**Xiangjun Pan:** Writing – original draft, Supervision, Investigation, Conceptualization. **Ruiyan Li:** Conceptualization. **Xinyu Xu:** Conceptualization. **Zehao Yu:** Writing – original draft, Investigation. **Shibo Liu:** Supervision, Methodology. **Dapeng Zeng:** Investigation. **Wenrui Qu:** Supervision. **Zhicheng Zhang:** Writing – original draft. **Hao Wang:** Writing – review & editing, Supervision, Methodology, Investigation, Funding acquisition. **Yanguo Qin:** Writing – review & editing.

## Declaration

Artificial intelligence tools were used only for the correction of spelling and grammatical errors in the manuscript. No AI participated in the creation of any original content presented in this work.

## Ethics approval and consent to participate

This article is a review of existing literature and does not contain any original studies with human participants or animals performed by any of the authors. Therefore, ethical approval and consent are not applicable.

## Fundings

This study was supported by funding from National Natural Science Foundation of China (82502914); 10.13039/501100001809National Natural Science Foundation of China (823B2059); the Key Project of the National Natural Science Foundation of China (U21A20390); Project of "Medical + X" Interdisciplinary Innovation Team of Norman Bethune Health Science Center of Jilin University (2024JBGS04); Jilin Provincial Key Laboratory of Orthopaedics, Department of Science and Technology of Jilin Province, Changchun, China; Beijing Tsinghua Changgung Hospital Fund (Grant No. 12024C01020).

## Declaration of competing interest

The authors declare that they have no known competing financial interests or personal relationships that could have appeared to influence the work reported in this review article.
