# Supplementary material for: A ‘three-axis synergy’ immunotherapeutic strategy for malignant bone tumors based on natural bioactives and bioactive materials
Source: Bioact Mater. 2026 Apr 29;64:73–93. doi: 10.1016/j.bioactmat.2026.04.031 (PMC13141613; doi:10.1016/j.bioactmat.2026.04.031)
Supplement: Multimedia component 1 [file mmc1.docx]

The literature search was conducted in PubMed, Web of Science Core Collection and Scopus, from database inception to March 2026.

In Pubmed: (osteosarcoma[Title/Abstract] OR "malignant bone tumor*"[Title/Abstract] OR "bone neoplasm*"[Title/Abstract]) AND (immunotherap*[Title/Abstract] OR "immune microenvironment"[Title/Abstract] OR "immune regulation"[Title/Abstract] OR "immune checkpoint*"[Title/Abstract]) AND ( "natural bioactive compound*"[Title/Abstract] OR "natural product*"[Title/Abstract] OR phytochemical*[Title/Abstract]) AND (biomaterial*[Title/Abstract] OR "bioactive material*"[Title/Abstract] OR scaffold*[Title/Abstract] OR hydrogel*[Title/Abstract] OR nanoparticle*[Title/Abstract]) AND ( "bone regeneration"[Title/Abstract] OR osteogenesis[Title/Abstract] OR "bone remodeling"[Title/Abstract] OR "bone repair"[Title/Abstract] OR osteoimmunology[Title/Abstract])

In Web of Science: TS=((osteosarcoma OR chondrosarcoma OR "Ewing sarcoma" OR "malignant bone tumor*" OR "bone neoplasm*" OR "bone cancer") AND (immunotherap* OR "immune microenvironment" OR "tumor immune microenvironment" OR "immunosuppressive microenvironment" OR "immune checkpoint*" OR "immune evasion" OR "immune regulation" OR TAM OR macrophage* OR "T cell*") AND ("natural bioactive compound*" OR "natural product*" OR phytochemical* OR "plant-derived compound*" OR flavonoid* OR polyphenol* OR alkaloid* OR terpenoid* OR curcumin OR resveratrol OR triptolide OR berberine OR quercetin OR icariin) AND (biomaterial* OR "bioactive material*" OR scaffold* OR hydrogel* OR nanoparticle* OR nanomaterial* OR microsphere* OR "delivery system*" OR implant* OR composite*))

In Scopus: TITLE-ABS-KEY ((osteosarcoma OR chondrosarcoma OR "Ewing sarcoma" OR "malignant bone tumor*" OR "bone neoplasm*" OR "bone cancer") AND (immunotherap* OR "immune microenvironment" OR "tumor immune microenvironment" OR "immunosuppressive microenvironment" OR "immune checkpoint*" OR "immune evasion" OR "immune regulation" OR TAM OR macrophage* OR "T cell*") AND ("natural bioactive compound*" OR "natural product*" OR phytochemical* OR "plant-derived compound*" OR flavonoid* OR polyphenol* OR alkaloid* OR terpenoid* OR curcumin OR resveratrol OR triptolide OR berberine OR quercetin OR icariin) AND (biomaterial* OR "bioactive material*" OR scaffold* OR hydrogel* OR nanoparticle* OR nanomaterial* OR microsphere* OR "delivery system*" OR implant* OR composite*))
